# Comparative Study of Neutral and Cationic Sn_2_H_2_: Toward Laboratory Detection of the Cation

**DOI:** 10.1021/acs.jpca.4c03220

**Published:** 2024-08-19

**Authors:** Samuel Biggerstaff, Nathaniel L. Kitzmiller, Justin M. Turney, Henry F. Schaefer

**Affiliations:** Center for Computational Quantum Chemistry, Department of Chemistry, University of Georgia, Athens, Georgia 30602, United States

## Abstract

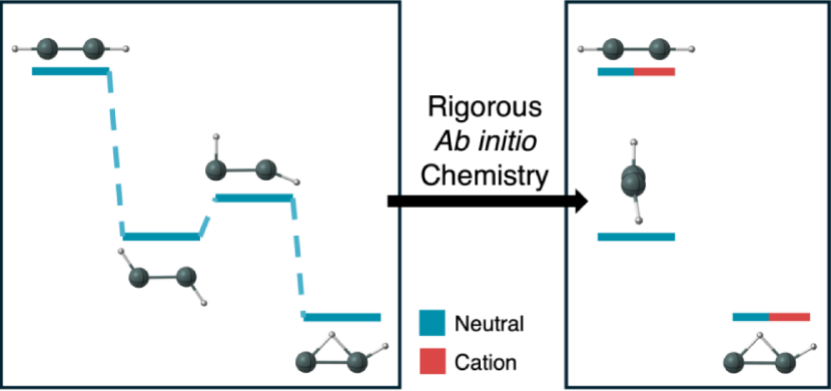

Group 14 M_2_H_2_ isomers (M = Si, Ge, Sn, and Pb) have attracted interest due to their radically differing electronic structures from acetylene. To better understand the Sn–H interactions of the neutral and cationic Sn_2_H_2_ structures, we present the most rigorous study of these systems to date. CCSD(T)/cc-pwCVTZ harmonic frequencies are presented as the first predictions for the neutral and cationic species to date. CCSDT(Q)/CBS relative energies are reported using the focal point approach, confirming the butterfly isomer as the global minimum on the potential energy surface for both the neutral and cationic species. In all, there exist 7 minima and 15 transition states. NBO analysis is also performed to elucidate the changes in bond order going from neutral to cation across all isomers of Sn_2_H_2_. Our results provide insights into the important Sn–H interaction and provide guidance for future work that may detect  in the laboratory for the first time.

## Introduction

Tin is a versatile metal whose chemistry has attracted interest because of its success in a range of applications, such as catalysis and hydrogen energy storage.^1−13^ Lewis acid zeolite-based catalysts such as Sn-BEA have produced greater than 90% yields in the conversion of certain renewable feedstocks, such as the conversion of triose sugars to lactic acid derivatives.^1,2^L-Zeolite-supported Pt/Sn catalysts have been shown to be uniquely effective for the dehydrogenation of isobutane, with up to 98% selectivity in the production of isobutene.^3^ Additionally, Sn-containing materials, such as La_2_Mg(Ni_0.095_Sn_0.05_)_9_,^4^ Nd_2_Sn_2_O_7_,^5^ LmNi_4.91_Sn_0.15_,^6,7^ and LaNi_5_, making small substitutions of Ni with Sn,^8−12^ have shown promise as hydrogen storage electrode alloys.

The aforementioned studies involving dehydrogenation catalysis and hydrogen energy storage materials highlight tin’s fascinating chemistry as it interacts with hydrogen. For example, in the production of the Pt/Sn catalyst presented by Cortright,^3^ hydrogen is introduced to doubly valent SnO to reduce the Sn^2+^ species to zero valency as Sn is alloyed to Pt. Additionally, the smaller size of the Sn atoms reduces the overall size of the Pt/Sn alloy surface in which Pt can act, decreasing the rate of isomerization and hydrogenolysis reactions and therefore increasing the selectivity of dehydrogenation. Small substitutions of Sn for Ni in LaNi_5_ increase the hydrogen storage capacity in materials with typically low hydrogen storage density.^8−12^ Sn-substituted materials have larger unit cell volumes compared to LaNi_5_,^11,12^ and the substitution improves stability proportionally for absorption–desorption cycling.^12^ Even simpler systems like a tin hydride of the form SnH_*x*_ have recently been found to display potential superconductivity under high pressure.^13^ Consequently, the study of simple systems containing only tin and hydrogen, that is, tin hydrides, may provide insights into electronic properties governing the broader field of tin chemistry.

Sn_2_H_2_ is an electronically interesting example of a tin hydride involved in an important segment of chemical history. The interest in this molecule stems from theoretical research involving the peculiar structure of the valence-isoelectronic Si_2_H_2_ molecule. Originally, Si_2_H_2_ was expected to exhibit an electronic structure similar to that of acetylene. However, through the inclusion of an effective core potential (ECP), the linear structure was determined to be instead a transition state with two imaginary vibrational frequencies.^14^ This discovery prompted a surge of research into Si_2_H_2_ and other similar molecules.^15−21^

**Figure 1:**
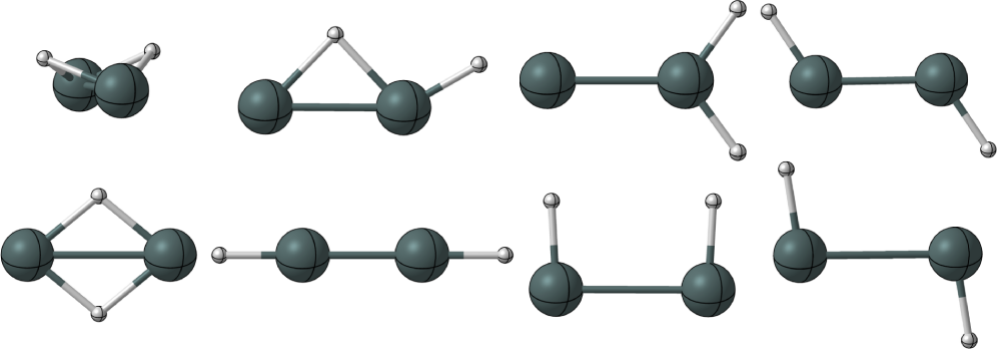
Quatlitative structures for the butterfly, monobridged, vinylidene-like, trans, planar dibridged, cis, and perptrans structures of Sn_2_H_2_.

Unlike acetylene, the global minimum of Si_2_H_2_ was not the linear structure, but rather a nonplanar structure with two hydrogen atoms bridging between the two Si atoms, referred to as the butterfly structure.^18^ Further investigations by Colegrove and Schaefer^19^ revealed an unprecedented minimum along the potential energy surface: the monobridged structure. This isomer contained one bridging hydrogen with other hydrogen bonded to one of the Si atoms. By further investigating this structure, Grev and Schaefer were able to report the first potential energy surface for the isomerization of Si_2_H_2_.^20^ Over the years of research dedicated to this molecule, the butterfly, monobridged, vinylidene-like, planar, cis, trans, and linear structures were identified as some of the primary stationary points that appeared along the potential energy surface (Figure 1). The butterfly isomer was found to be the global energy minimum; the monobridged, vinylidene-like, and planar trans isomers were found to be local minima, and the planar dibridged, cis, and linear structures were found to be transition states. As theoretical studies continued to investigate Si_2_H_2_, the unexpected monobridged structure was experimentally confirmed with microwave spectroscopy.^22^

At the time, several studies discussed the high sensitivity of the computations in relation to the basis set.^18−20^ The vibrational frequencies, in particular, were especially susceptible to changes in the method and basis. An example of this is the trans structure. Depending on the basis and level of theory, the trans species was previously reported to have one or two imaginary modes despite being presented as a minimum.

Investigations on Si_2_H_2_ continued, and some researchers became interested in other metal hydrides of similar structures. Thus, research appeared which began to explore the potential energy surface of other group 14 hydrides. Ge_2_H_2_^23−26^ was investigated thoroughly, and a qualitatively similar potential energy surface was determined. The butterfly was again reported as the global minima, with other stationary points prevalent in Si_2_H_2_ also appearing in Ge_2_H_2_. Furthermore, the vibrational frequencies were highly sensitive to the basis and level of theory. In the 1990 study by Grev, Deleeuw, and Schaefer,^23^ the trans structure displayed two imaginary modes. Both imaginary modes, a torsional *a*_u_ mode and a Ge–H stretching *b*_u_ mode, were determined to be artifacts of the method used (SCF/TZ2P). Experimentally, the butterfly isomer of Ge_2_H_2_ was confirmed by Wang and co-workers using matrix isolation.^24^ Pb_2_H_2_^25−27^ was investigated briefly, as well confirming similar electronic structures for the isomers along the potential energy surface, and it was potentially observed by Wang and Andrews^27^ with the same matrix isolation experiment used to identify the butterfly structure of Ge_2_H_2_.

**Figure 2:**
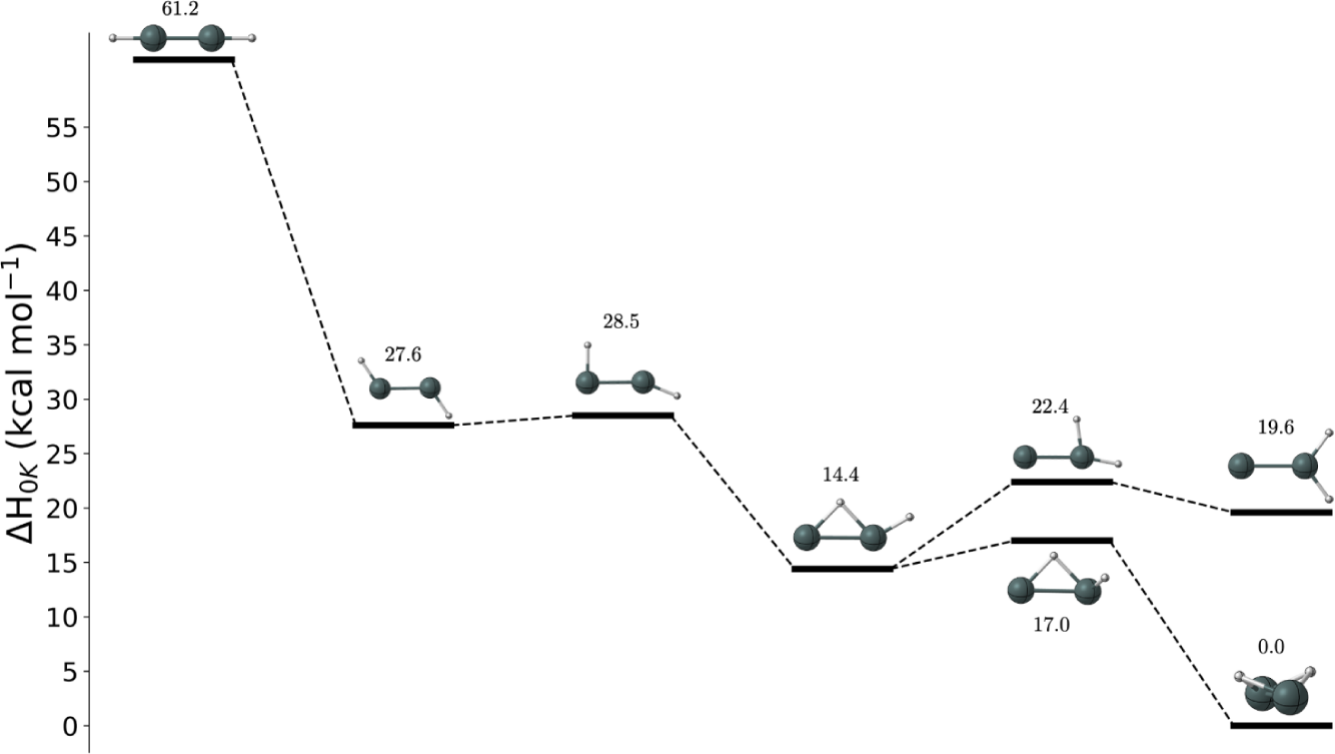
Potential energy surface for Sn_2_H_2_ with energies in kcal mol^–1^ determined by Nagase et al.^25^ with the MP2/6-311G(2d, 2p) method. Reproduced with permission from [^25^]. Copyright [2000] [Journal of Organometallic Chemistry].

In the unpublished 2010 study of the electronic structure of Sn_2_H_2_, geometries have been optimized up to the BP86/QZ4P level of theory with energies calculated at the CCSD(T)/aug-cc-pVQZ level of theory.^26^ Experimentally, Sn_2_H_2_ has been studied by Wang and co-workers, who successfully synthesized and found the vibrational spectra for the neutral form of Sn_2_H_2_ using matrix isolation.^28^ However, Wang and co-workers based their assignments on harmonic modes at the B3LYP/6-311++G**/SDD level of theory, and their assignments in the spectrum for In_2_H_2_ have been disputed.^29^ Furthermore, Nagase et al. reported a potential energy surface for Sn_2_H_2_ computed at the MP2/6-311G(2d, 2p) level of theory with zero-point energy corrections (Figure 2).^25^ This PES demonstrated structures and isomerization pathways qualitatively identical to the potential energy surface of Si_2_H_2_, Ge_2_H_2_, and Pb_2_H_2_, but it is unknown whether the underlying structure of this PES would remain intact under the investigation of higher level computations.

No experimental or theoretical results on the cation, Sn_2_H_2_^+^, are currently available. It would be fundamentally interesting to observe the resulting structures of a neutral species that already exhibits unusual stationary point geometries when it loses an electron. Given that other group 14 metal hydrides are extremely sensitive to the method and basis, it is imperative to investigate Sn_2_H_2_ with the most vigorous methods available. Thus, the present research investigates the electronic properties of Sn_2_H_2_ and its cation, Sn_2_H_2_^+^, and we present highly reliable structures and fundamental frequencies using rigorous *ab initio* methods.

## Computational Methods

### General Scheme

To account for the neutral and cationic forms of Sn_2_H_2_, restricted Hartree–Fock (RHF)^30^ was used for closed shell Sn_2_H_2_, and restricted open-shell Hartree–Fock (ROHF)^31^ and unrestricted Hartree–Fock (UHF)^32^ were used to treat open-shell Sn_2_H_2_^+^. ROHF was used in the geometry optimizations, energy calculations, and harmonic vibrational analysis, and UHF was used in the NBO analysis. All basis sets used in this study were downloaded from the Basis Set Exchange.^33−35^ Geometries, harmonic frequencies, and ionization energies were obtained using CFOUR 2.0.^36^ Energies were obtained using Molpro,^37−39^ Psi4,^40^ and MRCC,^41^ and the natural bond orders were obtained using NBO 7.0.^42^ Additionally, energies obtained from Molpro, Psi4, and MRCC were tested against energies obtained from CFOUR to ensure that differences in the software did not affect the results of the study.

### Geometries and Vibrational Frequencies

Stationary point geometries for the butterfly, monobridged, vinylidene-like, planar, cis, trans, and linear structures of Sn_2_H_2_ and Sn_2_H_2_^+^ were fully optimized with coupled cluster, single, double, and perturbative triple excitations, CCSD(T).^43−45^ The cc-pwCVXZ (X = D, T, and Q) basis sets^46,47^ (which will be referred to as XZ going forward) were used to describe all atoms. A 28-electron pseudo potential, or effective core potential (ECP), was used to treat Sn atoms for neutral and cation isomers and is designated by “–PP” in the name of the basis set.^48^ All other electrons, that is, those not included in the ECP, are fully correlated.

Harmonic vibrational frequencies and IR intensities were obtained in CFOUR by using finite differences of analytic gradients at the CCSD(T) level of theory and are reported at the CCSD(T)/TZ level of theory. Once geometries were obtained, center-of-mass dipole moments were recorded from geometry optimizations, and Wiberg bond index^49−51^ values and natural bond orders were calculated using NBO 7.0.

### Energies

Energies of the geometries optimized at the CCSD(T)/QZ level of theory were extrapolated to the complete basis set (CBS) limit using the focal point approach (FPA).^52−55^ Hartree–Fock (HF) energies were extrapolated using Feller’s three-point formula (1),^56^ and post-HF energies were estimated using Helgaker’s two-point formula (2):^57^
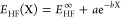
1
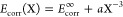
2Post-HF energies were extrapolated to the CBS limit up to the CCSD(T) level of theory. Higher order energy corrections were obtained at the CCSDT and CCSDT(Q) levels of theory with a DZ basis set, and the zero-point vibrational energies (ZPVE) were used for all isomers to correct energies with respect to their ground state vibrations at 0 K. Additionally, the ionization energy of the butterfly minimum was obtained with the EOMIP-CCSD/cc-pwCVQZ-PP method and basis.^58^

## Results and Discussion

### Geometries

**Figure 3:**
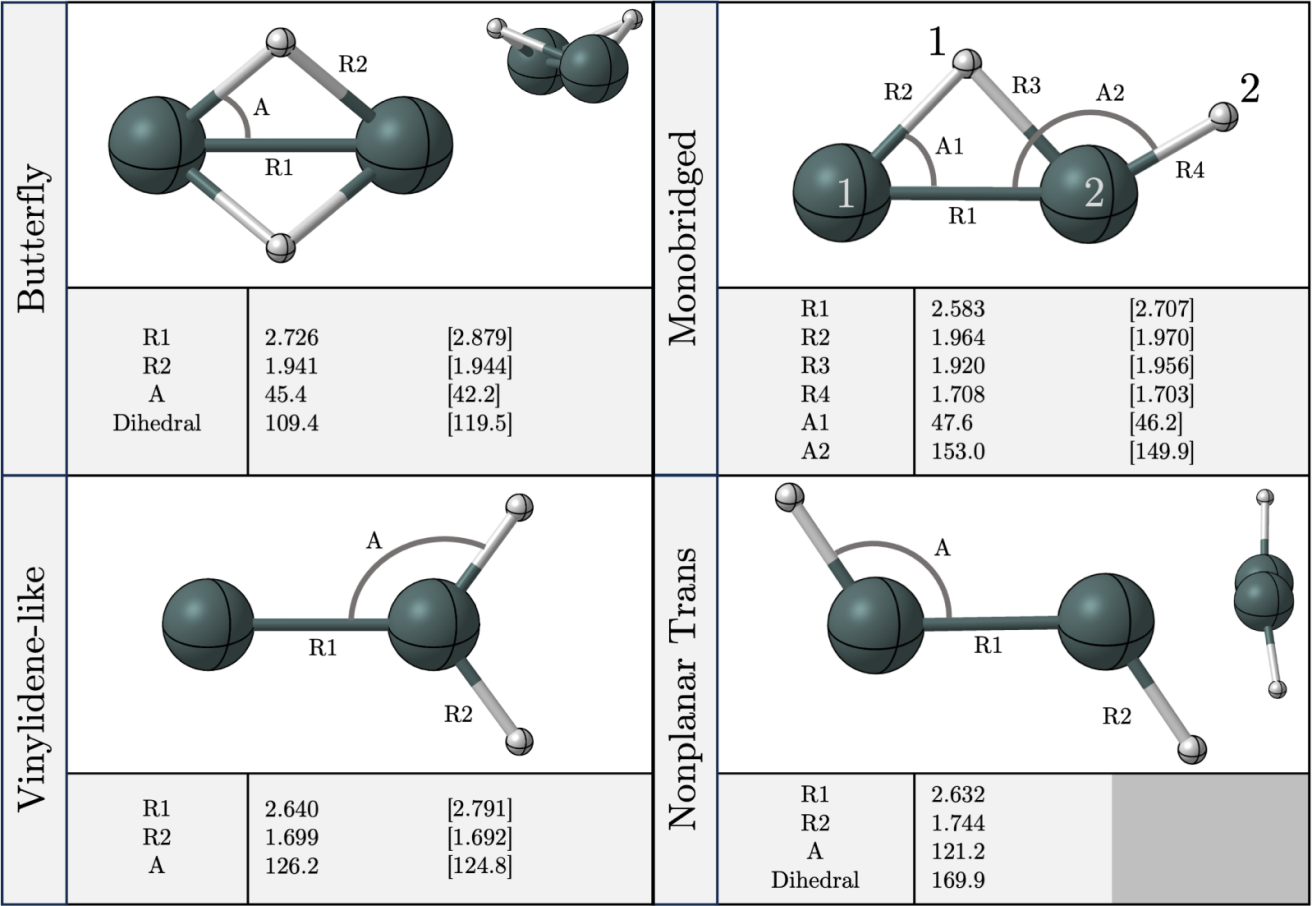
Equilibrium geometries predicted at the CCSD(T)/QZ level of theory for the butterfly, monobridged, vinylidene-like, and trans isomers. Bond lengths are in angstroms, and bond angles are in degrees. Values with brackets ([ ]) are associated with the cation species, and those without brackets are associated with the neutral species.

**Figure 4:**
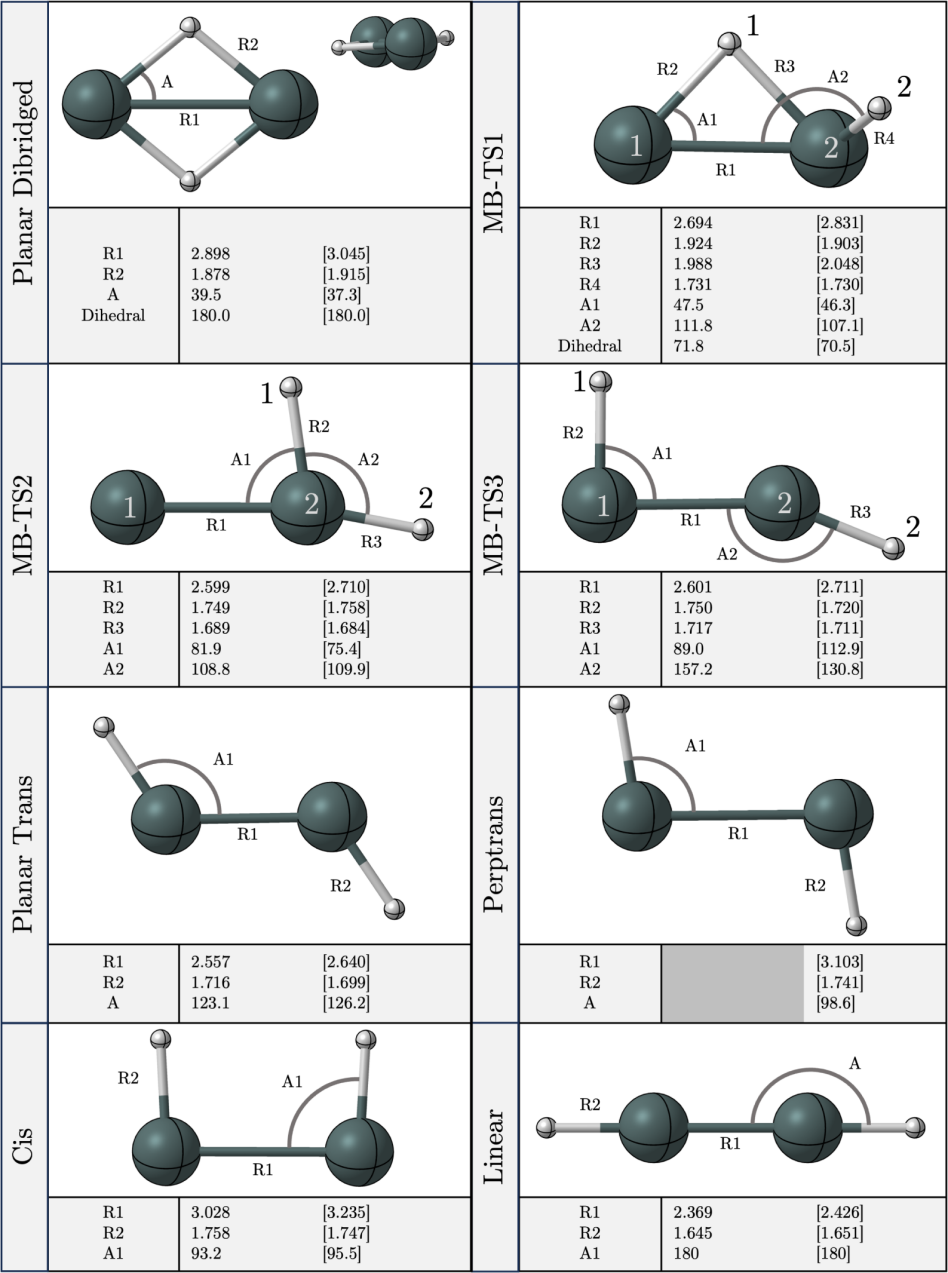
Stationary point geometries predicted for the planar dibridged, MB-TS1, MB-TS2, MB-TS3, trans-ts, perptrans, cis, and linear structures. All geometries are optimized at the CCSD(T)/QZ level of theory, except for the MB-TS structures which are optimized at CCSD(T)/TZ. Bond lengths are in angstroms, and bond angles are in degrees. Values with brackets ([ ]) are associated with the cation species, and those without brackets are associated with the neutral species.

Twelve stationary points were identified along the potential energy surface for neutral and cationic Sn_2_H_2_ with four minima (Figure 3) and eight transition states (Figure 4). Butterfly (C_2v_, neutral: ^1^A_1_, cation: ^2^A_1_), monobridged (C_s_, neutral: ^1^A^′^, cation: ^2^A^″^), vinylidene-like (C_2v_, neutral: ^1^A, cation: ^2^B_1_), and nonplanar trans (C_2_, neutral: ^1^A) structures were found to be minima, while planar dibridged (D_2h_, neutral: ^1^A_g_, cation: ^2^B_1u_), monobridged-like, perptrans (C_2h_, cation: ^2^A_g_), planar trans (C_2h_, neutral: ^1^A_g_, cation: ^2^A_u_), linear (D_∞h_, neutral: ^1^Π_g_, cation: ^2^Π_u_), and cis (C_2v_, neutral: ^1^A_1_, cation: ^2^A_1_) structures were found to be transition states. With the exception of the trans structures, the geometries of the neutral minima and transition states are in qualitative agreement with previous studies of Sn_2_H_2_.^25,26,28,59^ To differentiate between different trans structures, the molecule with the nearly perpendicular bond angles will be referred to as the perptrans structure, the molecule with a dihedral of 180^◦^ will be referred to as the planar trans structure, and the molecule with a dihedral of 169.9^◦^ will be referred to as the nonplanar trans structure. Additionally, three monobridged-like transition states have been investigated. In previous studies, similar monobridged-like structures have been identified for neutral M_2_H_2_ (M = Si, Ge, Sn, and Pb) as important transition states linking the monobridged minima to other minima along the potential energy surface.^20^ However, the monobridged-like transition states in this study have only been optimized to the CCSD(T)/TZ level of theory due to their lower symmetry and computational cost.

The majority of the structures identified were present in both the neutral and cationic forms, with the exception of the nonplanar trans and perptrans structures. The nonplanar trans structure was found as only a neutral molecule, and the perptrans structure was found only as a cation. Comparing the neutral and cation stationary points, several themes arise. First, the cation Sn–Sn bond is slightly elongated compared to the neutral bond. The largest difference between the bond lengths of the neutral and cation species occurs for the cis isomer (0.212 Å), and the smallest difference is found for the linear isomer (0.057 Å). Second, Sn–H bonds do not change as significantly compared to the difference in Sn–Sn bonds, suggesting that the majority of the lost electron density in the cation is removed from the Sn–Sn bond. Thus, many of the differences in bond angles between neutral and cation molecules arise from the lengthening of the Sn–Sn bond and relatively constant Sn–H bonds. Interestingly, in the butterfly structure, the dihedral angle changes by nearly 10^◦^, and the molecule becomes closer to the planar dibridged structure as it loses an electron. This effect was observed in Si_2_H_2_,^60^ but not to the same degree as Sn_2_H_2_.

Previously, the planar trans structure was identified as a local minimum along the potential energy surface for neutral Sn_2_H_2_.^25,26,59^ However, in this study, the planar trans structure was identified as a transition state, while the nonplanar trans structure was identified as a minimum. Geometrically, the neutral planar and nonplanar trans structures are not too dissimilar, with the greatest differences being their Sn–Sn bond lengths (2.557 and 2.632 Å, respectively) and dihedrals (180^◦^ and 169.9^◦^, respectively); however, their bonding structure, as shown later, is quite distinct from one another providing insights into their assignments as a transition state and minimum, respectively. The nonplanar trans structure was found only on the neutral potential energy surface, and no cation equivalent was identified in this study. Similarly, the perptrans transition state was only identified in the cation but does not seem to have a direct connection to the nonplanar trans minimum.

**Figure 5:**
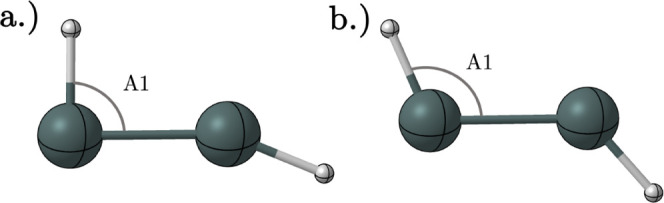
Geometries predicted at the CCSD(T)/TZ level of theory for the MB-TS3 (a) neutral and (b) cation structure.

The monobridged-like transition states (MB-TS1 (C_1_, neutral: ^1^A, cation: ^2^A), MB-TS2 (C_s_, neutral: ^1^A^′^, cation: ^2^A^″^), and MT-TS3 (C_s_, neutral: ^1^A^′^, cation: ^2^A^″^)) were investigated as well. MB-TS1 and MB-TS2, which have been previously identified as transition states between the monobridged and butterfly or vinylidene-like minima, respectively, were confirmed in this study.^25^ MB-TS3 was not observed to be a saddle point between the monobridged and planar trans structure. Since the planar trans structure was identified as a transition state, MB-TS3 cannot directly link the monobridged structure to the planar trans structure as a first-order saddle point. Additionally, the geometries of neutral and cation MB-TS3 vary the most compared to all other structures identified in this research (Figure 5).

The neutral structure (Figure 5a) looks more similar to the monobridged structure with an A1 bond angle of less than 90^◦^. In contrast, cation MB-TS3 (Figure 5b) displays a geometry that appears to be more similar to the planar trans structure with an A1 bond angle being greater than 90^◦^. Scans were performed to identify a transition state between the monobridged minimum and nonplanar trans minimum, but no such saddle points were found in this study.

### Relative Energies

Tables 1,2 show the CCSDT(Q)/CBS focal point energies and high-order coupled cluster corrections for the vinylidene-like neutral and cation structures. The remaining focal point tables can be found in Supporting Information. Excluding the planar and nonplanar trans structures, these tables demonstrate our rapid convergence to both the FCI and CBS limits, which validate the rigorous quantum chemistry methods used in this study.

**Table 1:** Focal Point Analysis Table for the Neutral Vinylidene-Like Isomer Relative to the Neutral Butterfly Isomer in kcal mol^–1^^a^^,^^b^

vinylidene-like neutral	HF	+δMP2	+δCCSD	+δ(T)	net
DZ	10.12	+9.24	–3.75	–0.05	[15.57]
TZ	10.85	+11.07	–5.15	+0.37	[17.14]
QZ	10.87	+11.35	–5.34	+0.48	[17.35]
5Z	10.87	+11.36	–5.31	+0.50	[17.43]
CBS limit	[10.87]	[+11.51]	[−5.45]	[+0.54]	[**17.46**]

a HF energies are extrapolated to the CBS limit using a three-point extrapolation formula, and post-HF energies are extrapolated using a two-point extrapolation method. Corrections for CCSDT, CCSDT(Q), and the zero-point vibration energy (ZPVE) are added to obtain the CCSDT(Q)/CBS energy.

b CCSD(T)/CBS + ΔZPVE + ΔT + ΔQ = CCSDT(Q)/CBS = **17.46** – 0.40 – 0.10 – 0.04 = 16.92

**Table 2:** Focal Point Analysis Table for the Cation Vinylidene-Like Isomer Relative to the Cation Butterfly Isomer in kcal mol^–1^^a^^,^^b^

vinylidene-like cation	HF	+δMP2	+δCCSD	δ(T)	net
DZ	3.67	+13.50	–3.24	+1.05	[14.99]
TZ	5.22	+14.58	–5.03	+1.52	[16.29]
QZ	5.29	+14.68	–5.30	+1.64	[16.31]
5Z	5.30	+14.60	–5.28	+1.67	[16.30]
CBS limit	[5.30]	[+14.75]	[−5.46]	[+1.71]	[**16.30**]

a HF energies are extrapolated to the CBS limit using a three-point extrapolation formula, and post-HF energies are extrapolated using a two-point extrapolation method. Corrections for CCSDT, CCSDT(Q), and the zero-point vibration energy (ZPVE) are added to obtain the CCSDT(Q)/CBS energy.

b CCSD(T)/CBS + ΔZPVE + ΔT + ΔQ = CCSDT(Q)/CBS = **16.30** – 0.08 + 0.16 – 0.13 = 16.25

The butterfly isomer was found to lie lowest in energy, followed by the monobridged, vinylidene-like, and trans isomer, as seen in Table 3. Energies for the monobridged-like transition states are given in Table 4. The order of energies did not differ between those of the cation and the neutral species. Additionally, there is no clear trend as to whether removing an electron from the system increases or decreases the relative energies. The planar dibridged transition state demonstrates the largest relative energy difference between the neutral and cation structure. The neutral planar dibridged structure lies 5.83 kcal mol^–1^ above the butterfly isomer, and the cation lies 1.66 kcal mol^–1^ above the butterfly isomer. When considering the change in the geometry, this energy difference appears reasonable. The planar dibridged structure acts as a transition state for the inversion of the butterfly structure. In the neutral form, the butterfly isomer lies farther away in its geometry from the planar isomer compared to its cationic counterpart. Therefore, the inversion barrier for the cation species is smaller, because its minimum lies closer to the geometry of the planar transition state. Additionally, the ionization energy of the butterfly global minimum was calculated to be 7.27 eV, a smaller ionization energy than that of C_2_H_2_ (11.49 eV).^61^

**Table 3:** Relative Energies at the CCSDT(Q)/CBS Approximation for the Butterfly, Planar, Monobridged, Vinylidene-Like, Perptrans, Cis, and Linear Isomers Given in kcal mol^–1^

	neutral	cation
butterfly	0.00	0.00
planar	5.83	1.66
monobridged	11.79	13.84
vinylidene-like	16.92	16.25
perptrans	- - -	25.32
planar trans	22.82	25.38
nonplanar trans	24.56	- - -
cis	28.56	27.53
linear	58.74	67.91

**Table 4:** Relative Energies at the CCSD(T)/cc-pwCVTZ Level of Theory with ZPVE Corrections for the Monobridged Transition States Relative to the Butterfly Isomer Given in kcal mol^–1^

	neutral	cation
MB-TS1	13.56	14.71
MB-TS2	19.01	20.22
MB-TS3	23.84	26.10

Across the majority of the focal point approximations, there is exceptional convergence toward the CCSDT(Q)/CBS limit. The relatively small high-order energy corrections of the focal point approach suggest that stationary points are sufficiently described by a single-reference determinant. As the methods increase in the excitation level, the high-order coupled cluster corrections converge rapidly with the largest correction for CCSDT being −0.52 kcal mol^–1^ in the cation linear transition state and the largest correction for CCSDT(Q) being +0.81 kcal mol^–1^ in the neutral nonplanar trans minimum. The neutral nonplanar trans minimum seems to be particularly sensitive to the level of theory and basis set. In the focal point table for the nonplanar trans minimum (Table 5), the HF/CBS energy is initially less than the HF/CBS energy of the neutral planar trans structure (Table 6); however, as the level of theory increases to CCSD(T), the energy of the nonplanar trans minimum becomes greater than that of the neutral trans structure. The swap in energy orderings also occurs when moving from the DZ to TZ basis. Additionally, both structures seem to be sensitive to the energy method and basis set, given their relatively large CCSDT and CCSDT(Q) correction. The corresponding planar trans cation (Table 7) has high-order energy corrections that are somewhat large compared to the rest of the structures but not to the degree of the neutral trans structures.

**Table 5:** Focal Point Analysis Table for the Neutral Nonplanar Trans Structure Relative to the Neutral Butterfly Isomer in kcal mol^–1^^a^^b^

nonplanar trans neutral	HF	+δMP2	+δCCSD	+δ(T)	net
DZ	26.87	+1.08	–2.44	–2.87	[22.63]
TZ	27.36	+2.52	–2.80	–2.66	[24.42]
QZ	27.41	+2.86	–2.77	–2.68	[24.83]
5Z	24.43	+2.91	–2.69	–2.69	[24.96]
CBS limit	[27.43]	[+3.08]	[−2.74]	[−2.69]	[**25.11**]

a HF energies are extrapolated to the CBS limit using a three-point extrapolation formula, and post-HF energies are extrapolated using a two-point extrapolation method. Corrections for CCSDT, CCSDT(Q), and thezero-point vibration energy (ZPVE) are added to obtain the CCSDT(Q)/CBS energy.

b CCSD(T)/CBS + ΔZPVE + ΔT + ΔQ = CCSDT(Q)/CBS = **25.11** – 1.25 – 0.11 + 0.81 = 24.56

**Table 6:** Focal Point Analysis Table for the Neutral Planar Trans Structure Relative to the Neutral Butterfly Isomer in kcal mol^–1^^a^^b^

planar trans neutral	HF	+δMP2	+δCCSD	+δ(T)	net
DZ	28.03	–3.43	+1.05	–2.52	[23.13]
TZ	28.31	–2.23	+0.49	–2.35	[24.23]
QZ	28.36	–2.06	+0.52	–2.37	[24.45]
5Z	28.37	–2.02	+0.59	–2.38	[24.56]
CBS limit	[28.38]	[−1.93]	[+0.54]	[−2.38]	[**24.60**]

a HF energies are extrapolated to the CBS limit using a three-point extrapolation formula, and post-HF energies are extrapolated using a two-point extrapolation method. Corrections for CCSDT, CCSDT(Q), and the zero-point vibration energy (ZPVE) are added to obtain the CCSDT(Q)/CBS energy.

b CCSD(T)/CBS + ΔZPVE + ΔT + ΔQ = CCSDT(Q)/CBS = **24.60** – 1.30 + 0.02 – 0.51 = 22.82

**Table 7:** Focal Point Analysis Table for the Cation Planar Trans Structure Relative to the Cation Butterfly Isomer in kcal mol^–1^^a^^b^

planar trans cation	HF	+δMP2	+δCCSD	+δ(T)	net
DZ	24.39	+4.27	–1.18	–1.28	[26.20]
TZ	25.53	+4.98	–2.28	–1.05	[27.18]
QZ	25.51	+5.05	–2.36	–1.03	[27.18]
5Z	25.52	+5.00	–2.29	–1.03	[27.20]
CBS limit	[25.52]	[+5.10]	[−2.41]	[−1.00]	[**27.20**]

a HF energies are extrapolated to the CBS Limit using a three-point extrapolation formula, and post-HF energies are extrapolated using a two-point extrapolation method. Corrections for CCSDT, CCSDT(Q), and the zero-point vibration energy (ZPVE) are added to obtain the CCSDT(Q)/CBS energy.

b CCSD(T)/CBS + ΔZPVE + ΔT + ΔQ = CCSDT(Q)/CBS = 27.20 – 1.39 – 0.17 – 0.26 = 25.38

### Potential Energy Surface

**Figure 6:**
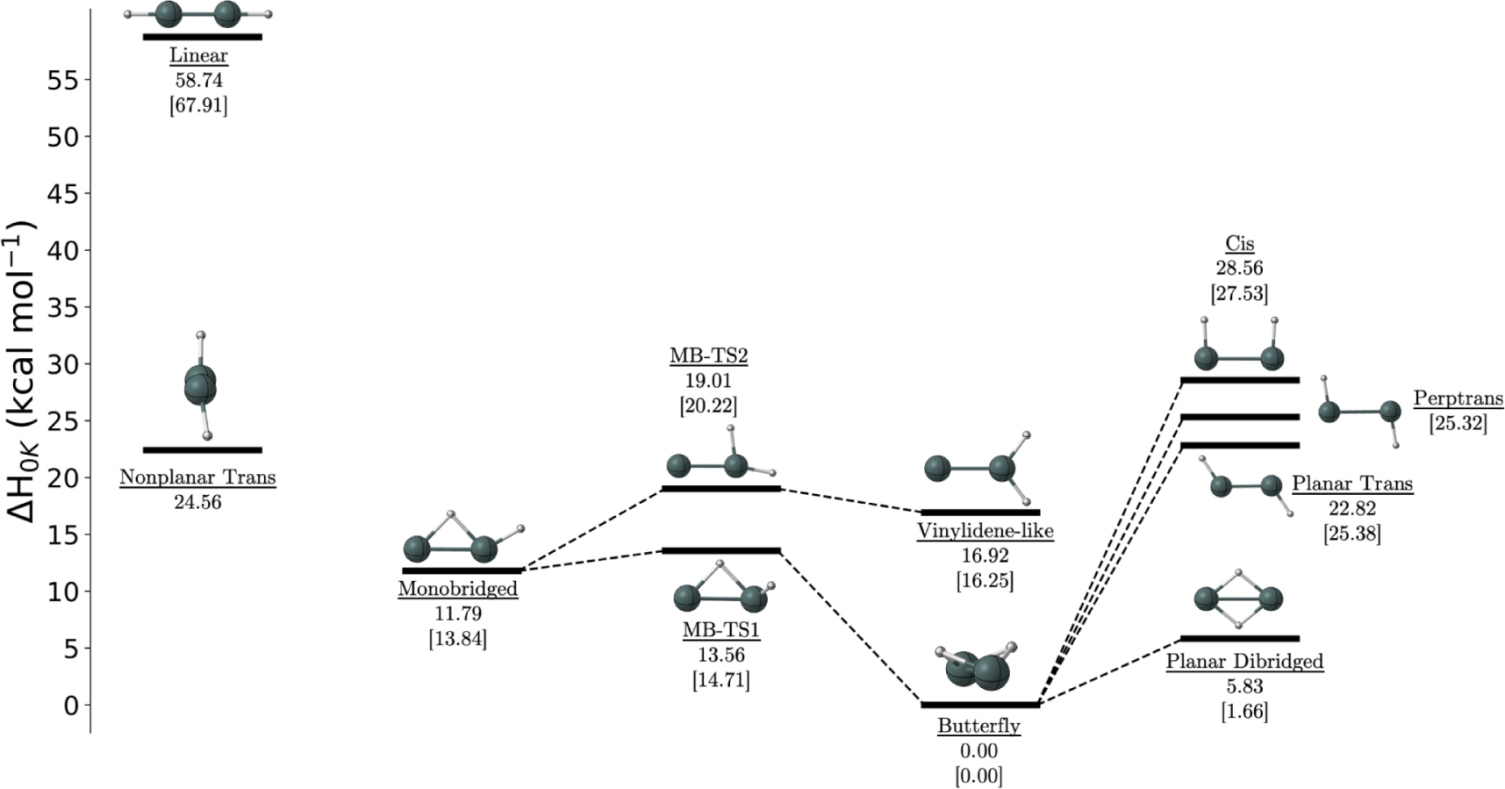
Potential energy surface for Sn_2_H_2_ with energies in kcal mol^–1^ calculated at the CCSDT(Q)/CBS limit with FPA with the exception of the MB-TS1, MB-TS2, and MB-TS3 structures, which were calculated at CCSD(T)/TZ with harmonic zero-point vibrational corrections. Energies without brackets represent neutral structures, and those with brackets ([ ]) represent cation structures.

Contrary to the work of Nagase et al., we found that the potential energy surface differs from that of other M_2_H_2_ (M = Si, Ge, or Pb) molecules (Figure 6). The primary difference is centered around the geometry of the nonplanar trans minimum. In the other M_2_H_2_ molecules, the trans minimum has a planar structure; however, we found that the trans minimum for Sn_2_H_2_ is nonplanar, therefore, changing the connectivity of minima and transition states.

Butterfly, monobridged, vinylidene-like, MB-TS1, and MB-TS2 remain qualitatively similar, with MB-TS1 bridging the monobridged and butterfly structures and MB-TS2 bridging the monobridged and vinylidene-like structures. MB-TS3, however, contains two imaginary modes and thus is not a first-order saddle point between two minima. Originally, MB-TS3 was thought to bridge the monobridged and planar trans minima, but due to the nonplanarity of the trans minimum, MB-TS3 no longer connects these structures. A saddle point between the nonplanar trans structure was investigated, but no such transition states were identified.

The planar trans structure was identified as a transition state in this study. The H-wagging motion of its imaginary mode seems to suggest that the structure would be connected to the nonplanar trans minimum, but the focal point analysis showed that the nonplanar trans minimum lies higher in energy than the planar trans transition state. Upon further investigation, the planar trans structure was found to connect to the butterfly structure with an intrinsic reaction path computation. Additionally, the planar dibridged, perptrans, and cis transition states were seen here to connect to the butterfly minimum.

### Vibrational Frequencies

The predicted CCSD(T)/TZ harmonic vibrational frequencies are presented in Tables 8–10. The majority of the infrared intensities are significantly small, but all cation minima contain at least one bright enough mode to be observed via infrared spectroscopy. The symmetric Sn–H stretch (1044 cm^–1^, *b*_1_) is the brightest mode for the butterfly structure. There has been no experimental analysis to date for the cation Sn_2_H_2_^+^, so there is no ability to compare our predicted modes to experiment. However, it is important to note that the ordering of modes is synonymous with that of the neutral species with relative intensities that match well.

**Table 8:** Harmonic Vibrational Frequencies in cm^–1^, Intensities in km mol^–1^, and Relative Intensities for the Butterfly, Monobirdged, Vinylidene-Like, and Nonplanar Trans Minima Are Given at the CCSD(T)/TZ Level of Theory^a^

	description	neutral		cation	
butterfly	sym Sn–H–Sn str. ω_1_ (*a*_1_)	1332.7	(18.2, 4.4)	1333.8	(5.4, 1.1)
	H wag ω_2_ (*a*_1_)	669.4	(45.5, 10.9)	594.7	(14.1, 2.7)
	Sn–Sn str. ω_3_ (*a*_1_)	193.6	(0.8, 0.2)	130.8	(0.0, 0.0)
	anti-sym. Sn–H str. ω_4_ (*a*_2_)	957.5	(0.0, 0.0)	1028.5	(0.0, 0.0)
	sym. Sn–H str. ω_5_ (*b*_1_)	1043.9	(**417.9**, 100.0)	1115.5	(**517.9**, 100.0)
	anti-sym Sn–H–Sn str. ω_6_ (*b*_2_)	1244.5	(76.9, 4.4)	1194.8	(75.7, 14.6)
monobridged	H2 str. ω_1_ (*a*^′^)	1840.8	(185.0, 100.0)	1878.4	(25.8, 18.3)
	H1 str. ω_2_ (*a*^′^)	1325.8	(121.0, 65.4)	1292.1	(47.6, 33.9)
	H1 bend ω_3_ (*a*^′^)	950.8	(137.1, 74.1)	921.4	(140.6, 100.0)
	H2 bend ω_4_ (*a*^′^)	414.1	(0.5, 0.3)	409.2	(0.3, 0.2)
	Sn–Sn str. ω_5_ (*a*^′^)	207.2	(3.1, 1.7)	173.8	(6.3, 4.5)
	H wag ω_6_ (*a*^″^)	133.9	(25.7, 13.9)	102.2	(3.0, 2.1)
vinylidene-like	sym Sn–H str. ω_1_ (*a*_1_)	1898.1	(137.1, 100.0)	1941.3	(23.8, 40.3)
	H scissor ω_2_ (*a*_1_)	696.3	(92.5, 67.5)	688.9	(59.0, 100.0)
	Sn–Sn str. ω_3_ (*a*_1_)	205.1	(3.3, 2.4)	167.0	(0.4, 0.8)
	H wag ω_4_ (*b*_1_)	236.1	(0.4, 0.3)	319.7	(4.4, 7.5)
	anti-sym Sn–H str. ω_5_ (*b*_2_)	1912.7	(136.7, 99.6)	1967.7	(40.3, 68.2)
	H rock ω_6_ (*b*_2_)	195.4	(19.1, 13.9)	219.8	(9.1, 15.5)
nonplanar trans	sym Sn–H str. (*a*)	1810.9	(1.37, 0.4)	- - -	- - - - - -
	sym Sn–Sn–H bend (*a*)	407.1	(2.46, 0.7)	- - -	- - - - - -
	H wag (*a*)	352.8	(0.11, 0.0)	- - -	- - - - - -
	Sn–Sn str. (*a*)	159.0	(0.11, 0.0)	- - -	- - - - - -
	anti-sym Sn–H str. (*b*)	1821.1	(379.1, 100.0)	- - -	- - - - - -
	anti-sym Sn–Sn–H bend (*b*)	191.2	(11.3, 3.0)	- - -	- - - - - -

a Bolded values indicate intensities greater than 400 km mol^–1^.

**Table 9:** Harmonic Vibrational Frequencies in cm^–1^, Intensities in km mol^–1^, and Relative Intensities for the Planar, Linear, Cis, Perptrans, and Trans Transition States Are Given at the CCSD(T)/TZ Level of Theory^a^

	description	neutral		cation	
planar dibridged	sym Sn–H–Sn str. ω_1_ (*a*_g_)	1405.8	(0.0, 0.0)	1361.0	(0.0, 0.0)
	Sn–Sn str. ω_2_ (*a*_g_)	180.8	(0.0, 0.0)	176.4	(0.0, 0.0)
	Anti-sym Sn–H str. ω_3_ (*b*_1g_)	1359.2	(0.0, 0.0)	1266.2	(0.0, 0.0)
	anti-sym Sn–H–Sn str. ω_4_ (*b*_2u_)	1184.8	(81.2 13.0)	1112.0	(70.0, 9.2)
	sym Sn–H str. ω_5_ ()	1340.0	(**625.5**, 100.0)	1297.6	(**757.9**, 100.0)
	H wag ω_5_ (*b*_1u_)	437.7*i*	(10.7, 1.7)	312.9*i*	(2.9, 0.4)
linear	sym Sn–H str. ω_1_ ()	2038.6	(0.0, 0.0)	2031.5	(0.0, 0.0)
	Sn–Sn str. ω_2_ ()	287.3	(0.0, 0.0)	283.6	(0.0, 0.0)
	anti-sym Sn–H str. ω_3_ ()	2047.1	(0.2, 1.9)	2050.0	(105.1, 100.0)
	sym Sn–Sn–H bend ω_4_ (π_u_)	341.2	(9.6, 100.0)	314.6	(16.6, 15.8)
	sym Sn–Sn–H bend ω_5_ (π_u_)	341.2	(9.6, 100.0)	314.6	(16.6, 15.8)
	anti-sym Sn–Sn–H bend ω_6_ (π_g_)	844.3*i*	(0.0, 0.0)	921.7*i*	(0.0, 0.0)
	anti-sym Sn–Sn–H bend ω_7_ (π_g_)	844.3*i*	(0.0, 0.0)	921.7*i*	(0.0, 0.0)
cis	sym. Sn–H str. ω_1_ (*a*_1_)	1751.0	(**547.0**, 100.0)	1806.9	(330.6, 100.0)
	sym. Sn–Sn–H bend ω_2_ (*a*_1_)	363.4	(9.8, 1.8)	301.9	(0.0, 0.0)
	Sn–Sn str. ω_3_ (*a*_1_)	195.0	(0.8, 0.2)	99.0	(0.0, 0.0)
	anti-sym Sn–H str. ω_4_ (*b*_2_)	1716.1	(**459.8**, 84.1)	19.1	(0.1, 0.0)
	anti-sym Sn–Sn–H bend ω_5_ (*b*_2_)	459.3	(49.9, 9.1)	1792.6	(30.8, 9.3)
	H scissor ω_6_ (*a*_2_)	494.5*i*	(0.0, 0.0)	281.7*i*	(0.0, 0.0)
perptrans	sym Sn–H str. ω_1_ (*a*_g_)	- - -	- - - - - -	1812.9	(0.0, 0.0)
	sym Sn–Sn–H str. ω_2_ (*a*_g_)	- - -	- - - - - -	449.5	(0.0, 0.0)
	Sn–Sn str. ω_3_ (*a*_u_)	- - -	- - - - - -	106.9	(0.0, 0.0)
	anti-sym Sn–H str. ω_4_ (*b*_u_)	- - -	- - - - - -	1821.5	(319.2, 100.0)
	anti-sym Sn–Sn–H bend ω_5_ (*b*_u_)	- - -	- - - - - -	235.9	(0.9, 0.3)
	H wag ω_6_ (*a*_g_)	- - -	- - - - - -	283.3*i*	(0.8, 0.2)
planar trans	sym Sn–H str. ω_1_ (*a*_g_)	1827.6	(0.0, 0.0)	1841.6	(0.0, 0.0)
	sym Sn–Sn–H bend ω_2_ (*a*_g_)	479.8	(0.0, 0.0)	476.1	(0.0, 0.0)
	Sn–Sn str. ω_3_ (*a*_g_)	208.2	(0.0, 0.0)	152.4	(0.0, 0.0)
	anti-sym Sn–H str. ω_4_ (*b*_u_)	1840.3	(326.4, 100.0)	1850.6	(98.4, 100.0)
	anti-sym Sn–Sn–H bend ω_5_ (*b*_u_)	154.9	(14.1, 4.3)	123.7	(30.5, 31.0)
	H wag ω_6_ (*a_u_)*	277.8*i*	(120.3, 37.0)	236.9*i*	(16.6, 16.9)

a Bolded values indicate intensities greater than 400 km mol^–1^.

**Table 10:** Harmonic Vibrational Frequencies in cm^–1^, Intensities in km mol^–1^, and Relative Intensities for the MB-TS1, MB-TS2, and MB-TS3 Transition States Are Given at the CCSD(T)/TZ Level of Theory

	description	neutral		cation	
MB-TS1	Sn2–H2 str. ω_1_ (*a*)	1784.7	(195.7, 100.0)	1817.8	(78.4, 34.7)
	Sn1–H1 str. ω_2_ (*a*)	1269.7	(127.3, 65.0)	1312.4	(166.1, 73.5)
	Sn2–H1 str. ω_3_ (*a*)	900.5	(151.4, 77.4)	849.1	(226.1, 100.0)
	H scissor ω_4_ (*a*)	578.6	(25.6, 13.1)	574.4	(7.8, 3.4)
	Sn–Sn str. ω_5_ (*a*)	210.9	(0.5, 0.3)	154.4	(0.2, 0.1)
	H wag ω_6_ (*a*)	191.9*i*	(19.6, 10.0)	128.7*i*	(12.6, 5.6)
MB-TS2	Sn2–H2 str. ω_1_ (*a*^′^)	1934.4	(18.7, 44.1)	1952.4	(17.5, 29.9)
	Sn2–H1 str. ω_2_ (*a*^′^)	1753.5	(42.4, 100.0)	1719.4	(58.6, 100.0)
	H scissor ω_3_ (*a*^′^)	665.0	(41.3, 97.4)	646.4	(41.9, 71.5)
	Sn–Sn str. ω_4_ (*a*^′^)	239.9	(2.1, 5.0)	192.9	(1.7, 2.9)
	H wag ω_5_ (*a*^″^)	281.5	(0.4, 1.0)	220.9	(0.0, 0.0)
	H rock ω_6_ (*a*^′^)	263.9*i*	(2.0, 4.7)	327.5*i*	(2.5, 4.3)
MB-TS3	Sn2–H2 str. ω_1_ (*a*^′^)	1825.9	(221.8, 100)	1871.4	(50.3, 100)
	Sn1–H1 str. ω_2_ (*a*^′^)	1730.6	(167.5, 75.5)	1847.3	(33.7, 67.0)
	H rock ω_3_ (*a*^′^)	486.8	(16.0, 7.2)	477.4	(0.4, 0.8)
	Sn–Sn str. ω_4_ (*a*^′^)	202.5	(0.2, 0.1)	170.7	(0.7, 1.4)
	H scissor ω_5_ (*a*^′^)	203.8*i*	(5.0, 2.3)	109.5*i*	(19.0, 37.8)
	H wag ω_6_ (*a*^″^)	76.0*i*	(26.2, 11.8)	189.6*i*	(11.0, 21.9)

With the exception of the trans structures, the identification of minima and transition states agrees with previous studies of similar M_2_H_2_ molecules including Sn_2_H_2_. However, the planar trans structure was found to be a transition state with an imaginary mode, while the nonplanar trans structure was found to be a minima. This diverges from previous studies which identified the planar trans structure as the minima.^18,19,23^ The imaginary mode in the planar trans structure (228*i* cm^–1^, *a*_u_) is a H-wagging mode indicating that the nearby minima are most likely not planar. Manually searching the potential energy surface at the CCSD(T)/DZ and CCSD(T)/TZ levels of theory revealed a slight decrease in energy, leading to the discovery of the nonplanar trans structure. However, FPA determined that these minima lie 1.74 kcal mol^–1^ higher in energy than the planar trans structure. The harmonic vibrational frequencies of the nonplanar trans structure do not contain any imaginary modes, suggesting that this structure does exist as a minimum. Further attempts at finding minima connecting to the planar trans transition state identified the butterfly as the corresponding minimum in both directions of the planar trans structure’s imaginary mode.

For most structures, there is excellent convergence between different basis set sizes; however, the cis cation is an exception to this observation. With the TZ basis set in Table 9, the cis cation contains a very low lying mode (19.1 cm^–1^). When the harmonic frequency calculations of different basis sets are compared, this mode becomes an imaginary mode as the basis set size increases (Table 11). Such a sensitivity to the basis set is a characteristic which has been shown for other isomers, particularly for the trans structure in Si_2_H_2_.^18−20^ In contrast, this low lying mode and convergence toward two imaginary modes are not observed in the neutral form of the cis structure. Instead, the neutral molecule shows excellent convergence toward its identity as a first-order saddle point.

**Table 11:** Harmonic Vibrational Frequencies in cm^–1^ for the Cis Cation Transition State Are Given at the CCSD(T)/DZ, CCSD(T)/TZ, and CCSD(T)/QZ Level of Theory

	description	DZ	TZ	QZ
cis	sym. Sn–H str. ω_1_ (*a*_1_)	1805.9	1806.9	1809.8
	sym. Sn–Sn–H bend ω_2_ (*a*_1_)	300.9	301.9	300.2
	Sn–Sn str. ω_3_ (*a*_1_)	95.1	99.0	99.6
	anti-sym Sn–H str. ω_4_ (*b*_2_)	76.1	19.1	74.4*i*
	anti-sym Sn–Sn–H bend ω_5_ (*b*_2_)	1792.4	1792.6	1795.2
	H scissor ω_6_ (*a*_2_)	266.2*i*	281.7*i*	286.4*i*

Additionally, three monobridged transition states were evaluated because of their importance to the potential energy surface of Sn_2_H_2_ (Table 10). MB-TS1 and MB-TS2 were transition states, which agreed with the previous studies on similar molecules. MB-TS1 bridges the monobridged and butterfly minima, and MB-TS2 bridges the monobridged and vinylidene-like minima. MB-TS3, however, does not agree with previous research, where the structure now displays two imaginary modes. In smaller M_2_H_2_ molecules, a structure similar to MB-TS3 would bridge the monobridged and the trans minima, but the nonplanarity of the trans minimum does not allow for this pathway, and MB-TS3 does not appear as a first-order saddle point.

In the experimental analysis of the neutral species by Andrews and co-workers,^28^ they identified two peaks in their infrared spectrum that were associated with the butterfly structure of Sn_2_H_2_. They used the B3LYP/6-311++G**/SDD level of theory to assist in elucidating the nature of the vibrations. The first of these peaks was in a bright mode (913 cm^–1^). Wang and co-workers assigned this mode to an antisymmetric Sn–H–Sn stretch (970 cm^–1^, *b*_2_). Our analysis, however, predicts the *b*_2_ mode to lie higher in frequency (1237 cm^–1^) and to have a lower relative intensity (9.7). However, the symmetric Sn–H stretching mode (1044 cm^–1^, *b*_1_) lies closer in frequency to the observed vibration and has a greater relative intensity (100.0). The second peak identified was a much weaker vibration (1118 cm^–1^) which was assigned as the antisymmetric Sn–H–Sn stretching mode (1176 cm^–1^, *b*_1_). In contrast, our analysis identified the second antisymmetric mode (958 cm^–1^, *a*_2_) as having different symmetry and not being IR visible. The presence of two antisymmetric Sn–H–Sn stretching modes with different symmetries suggests a misprint in Wang and co-workers original paper, and according to the results of the present study, it would appear that the first brighter mode is the symmetric Sn–H stretching mode (1044 cm^–1^, *b*_1_) with an intensity of 418 km mol^–1^, and the second, weaker mode is the antisymmetric Sn–H–Sn streching mode (1244.5 cm^–1^, *b*_2_) with an intensity of 77 km mol^–1^.

### NBO

Natural bond order (NBO) analysis was used to determine the orbitals, natural bond orders, and Wiberg bond index values for neutral and cation molecules (Table 12). Among each of the isomers, the cation Sn–Sn bond order decreases by roughly 0.5, further demonstrating that the electron lost in the cation is mostly being removed from the Sn–Sn bond. The Sn–H bond orders did not change significantly, where the largest difference using the Wiberg bond index was 0.09 in the Sn–H interaction in the cis isomer and the largest difference using natural bond orders was 0.23 in the planar trans structure. In the linear isomer, there was a H–H interaction with a 0.11 Wiberg bond index value, but this was not seen for the natural bond orders.

**Table 12:** Wiberg Bond Index Values Obtained from NBO 7.0 for the Butterfly, Monobridged, Vinylidene-Like, Nonplanar Trans, Planar Dibridged, MB-TS1, MB-TS2, MB-TS3, Planar Trans, Pertrans, Cis, and Linear Structures Which Were Optimized to the CCSD(T)/QZ Level of Theory

		Wiberg		NBO	
		neutral	cation	neutral	cation
butterfly	Sn–Sn	1.11	0.64	1.27	0.62
	Sn–H	0.35	0.38	0.43	0.46
	H–H	0.00	0.00	0.00	0.00
monobridged	Sn–Sn	1.82	1.13	2.05	1.50
	Sn1–H1	0.40	0.38	0.39	0.43
	Sn2–H1	0.52	0.48	0.47	0.45
	Sn2–H2	0.89	0.89	0.91	0.87
	H–H	0.00	0.00	0.06	0.00
vinylidene-like	Sn–Sn	1.85	1.15	1.99	1.38
	Sn–H	0.92	0.91	0.96	0.93
	H–H	0.01	0.01	0.00	0.00
nonplanar trans	Sn–Sn	2.17	- - -	2.18	- - -
	Sn–H	0.88	- - -	0.97	- - -
	H–H	0.00	- - -	0.00	- - -
planar dibridged	Sn–Sn	1.25	0.72	1.27	0.62
	Sn–H	0.42	0.38	0.43	0.46
	H–H	0.05	0.04	0.00	0.00
MB-1	Sn–Sn	1.29	0.89	1.96	1.27
	Sn1–H1	0.48	0.48	0.47	0.48
	Sn1–H2	0.04	0.04	0.00	0.00
	Sn2–H2	0.89	0.89	0.86	0.92
	Sn2–H1	0.40	0.34	0.41	0.43
	H1–H2	0.00	0.00	0.00	0.00
MB-2	Sn–Sn	1.85	1.14	2.00	1.44
	Sn1–H1	0.06	0.06	0.00	0.00
	Sn1–H2	0.05	0.05	0.00	0.00
	Sn2–H2	0.91	0.92	0.95	0.93
	Sn2–H1	0.88	0.86	0.94	0.91
	H1–H2	0.00	0.00	0.00	0.00
MB-3	Sn–Sn	1.84	1.33	1.90	1.48
	Sn1–H1	0.84	0.87	0.90	0.89
	Sn1–H2	0.05	0.05	0.00	0.00
	Sn2–H2	0.86	0.87	0.95	0.94
	Sn2–H1	0.08	0.06	0.00	0.00
	H1–H2	0.00	0.00	0.00	0.00
planar trans	Sn–Sn	2.15	1.43	2.50	1.76
	Sn–H	0.88	0.87	0.75	0.98
	H–H	0.00	0.00	0.00	0.00
perptrans	Sn–Sn	- - -	0.66	- - -	0.97
	Sn–H	- - -	0.84	- - -	0.99
	H–H	- - -	0.00	- - -	0.00
cis	Sn–Sn	0.99	0.58	0.96	0.97
	Sn–H	0.93	0.84	0.98	0.98
	H–H	0.03	0.00	0.02	0.00
linear	Sn–Sn	2.95	2.41	2.90	2.35
	Sn–H	0.98	0.94	0.95	0.93
	H–H	0.11	0.01	0.00	0.00

The changes in the bond orders of the isomers between their neutral and cation forms may be correlated with the changes in geometries. As an electron is removed from each neutral structure, the Sn–Sn bond length increases and the bond order decreases, supporting the argument that the majority of the electron that is lost from the neutral species is removed from the vicinity of the Sn–Sn bond. This trend can also be observed in the comparison of the neutral and cation species of Si_2_H_2_.^60^

**Figure 7:**
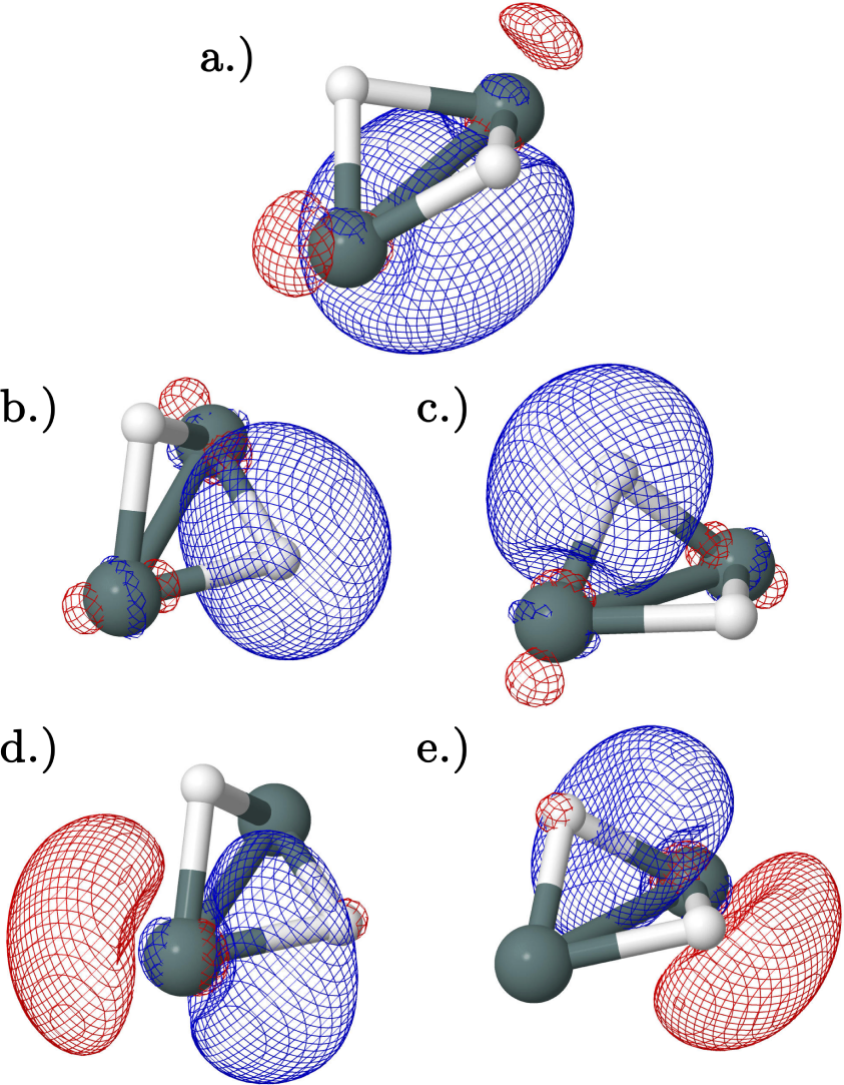
Natural bonding orbitals obtained from NBO analysis of the CCSD(T)/QZ geometries of the butterfly cation and neutral minima: (a) Sn–Sn bonding orbital with primarily π character (HOMO/SOMO). (b,c) Degenerate Sn–H bonding orbitals. (d,e) Degenerate lowest unoccupied molecular orbitals (LUMO). Orbitals (b,d) and orbitals (c,e) have second-order perturbation interaction energies of 161 kcal mol^–1^ and 67 kcal mol^–1^ in the neutral and cation, respectively. The cation and neutral are represented in one image as their orbitals are qualitatively similar.

Additionally, the orbitals of the neutral and cationic species of Sn_2_H_2_ were investigated. All HOMO, LUMO, SOMO, bonding, and important interaction orbitals are shown in Supporting Information. Overall, the NBO orbital analysis agrees with the research of Frenking and co-workers^59^ for the neutral species, with the exception of the trans and monobridged structures. In the butterfly structure (Figure 7), there are a Sn–Sn single bond (Figure 7a), two Sn–H single bonds Figure 7b,c, two donor–acceptor bonds formed between an empty π orbital (Figure 7d,e), and a Sn–H bond on an adjacent Sn atom. This donor–acceptor interaction gives a notably large stabilizing second-order perturbation energy of 161 kcal mol^–1^ and 67 kcal mol^–1^ in the neutral and cationic species, respectively, resulting in the two bridging hydrogens. This is responsible for the unusual nonplanar butterfly structure where the hydrogens are tilted toward the adjacent Sn atom. The other Sn_2_H_2_ structures behave as discovered in the analysis of Lein et al.,^59^ except for the trans and monobridged structures.

**Figure 8:**
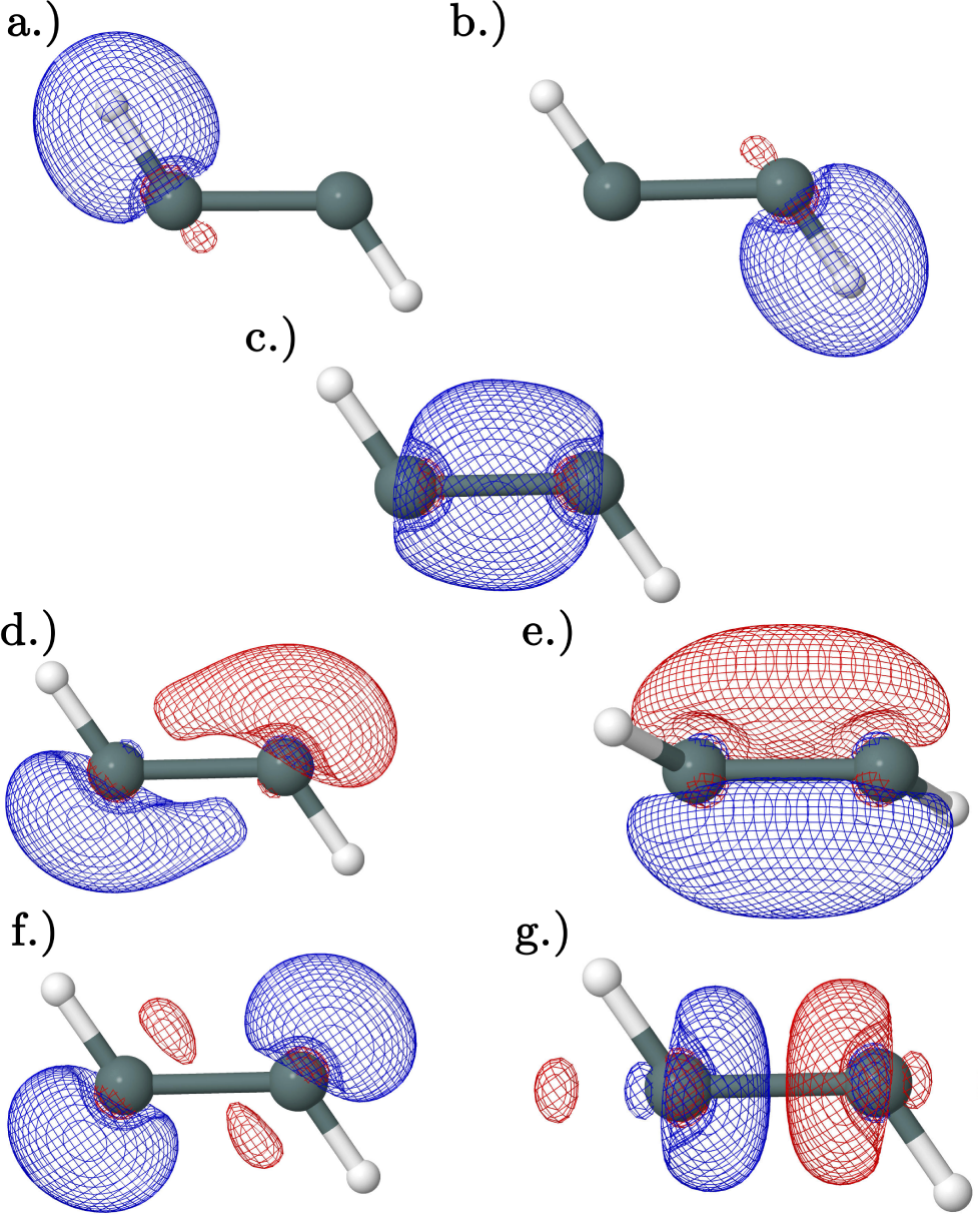
Natural bonding orbitals obtained from NBO analysis of the CCSD(T)/QZ geometry of the neutral planar trans transition state: (a,b) degenerate Sn–H bonding orbitals. (c) Sn–Sn bonding orbital with primarily σ character. (d) Sn–Sn bonding orbital with primarily π character. (e) HOMO Sn–Sn bonding orbital with primarily π character. (f) LUMO. (g) Important bonding interaction orbital. Orbitals (c,f) have a second-order perturbation interaction energy of 103 kcal mol^–1^, and orbitals (d,g) have a second-order perturbation interaction energy of 87 kcal mol^–1^.

Unlike Lein et al.,^59^ this study found that the trans minima are not planar, containing a small torsion (τ = 169.9°). A planar trans structure does exist, but it was found to be a transition state according to its harmonic vibrational analysis. Beginning with the planar transition state, NBO analysis suggests that there are three bonding (Figure 8a–c) orbitals with no donor–acceptor-type interactions and a natural bond order of 2.50 in the neutral species indicating that resonance has a noteworthy impact on bonding. The neutral species contains two pairs of interacting orbitals: Figure 8c–f (103 kcal mol^–1^) and Figure 8d–g (87 kcal mol^–1^), and the HOMO (Figure 8e) has no strong interactions.

**Figure 9:**
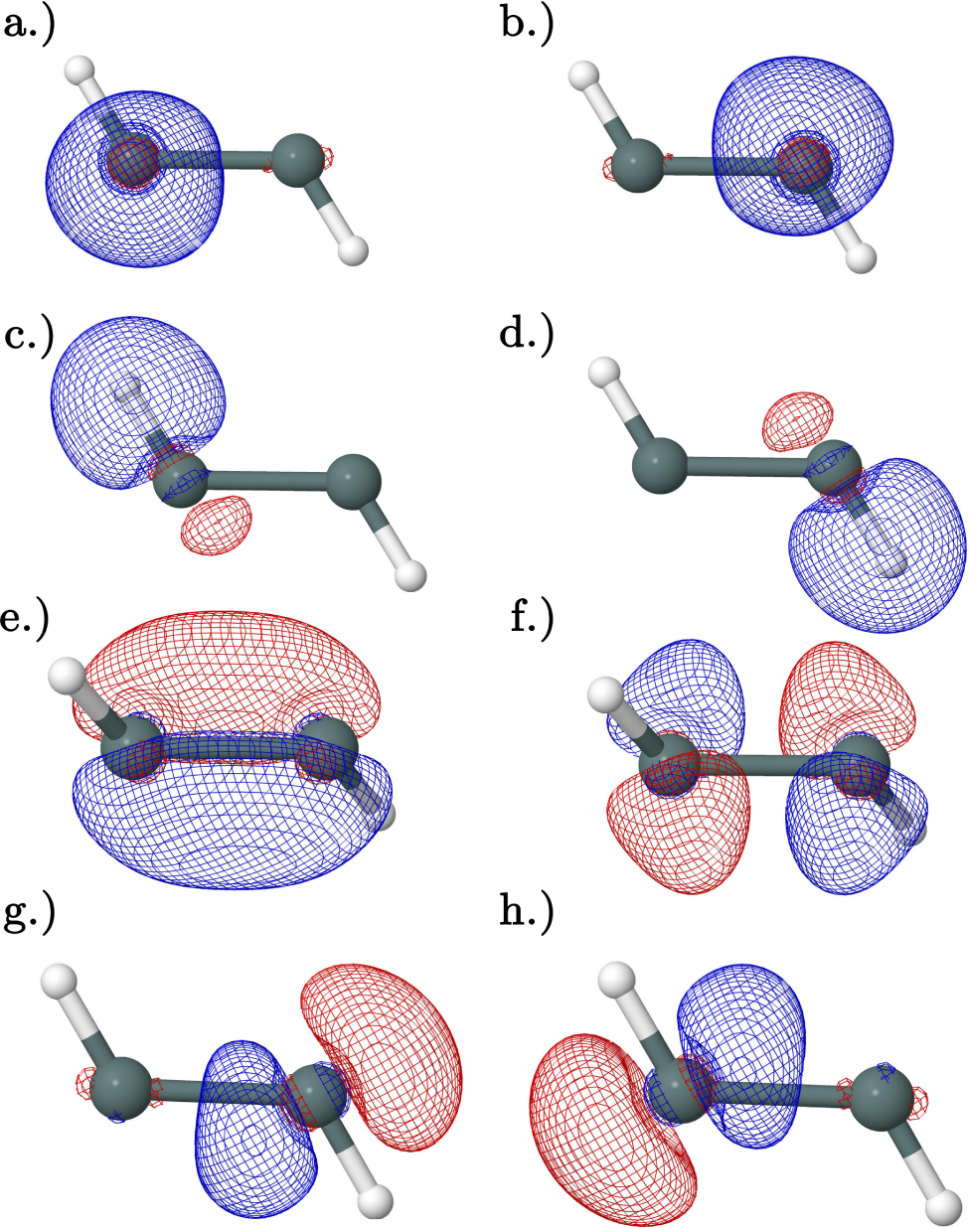
Natural bonding orbitals obtained from NBO analysis of the CCSD(T)/QZ geometry of the cation planar trans transition state: (a,b) degenerate Sn lone pair orbitals. (c,d) Degenerate Sn–H bonding orbitals. (e) HOMO Sn–Sn bonding orbital with primarily π character. (f) LUMO. (g,h) Important bonding interaction orbitals. Orbitals (a,g) and orbitals (b,h) have a second-order perturbation interaction energy of 46 kcal mol^–1^.

Unlike other Sn_2_H_2_ structures which have similar natural bonding orbitals between the neutral and cation, the planar trans cation has qualitatively different orbitals compared to its neutral counterpart (Figure 9). The cation contains two Sn–H single bonding orbitals (Figure 9c,d), no Sn–Sn bonding orbitals with the σ character, and only one Sn–Sn bonding orbital with the π character (Figure 9e). Additionally, the interacting orbitals differ with two pairs of degenerate interacting orbitals increasing the overall bond order: Figure 9a–g and Figure 9b–h (45.60 kcal mol^–1^), and the HOMO (Figure 9f) has no strong interactions. The cation planar trans structure fits the qualitative picture presented by Lein et al.^59^

**Figure 10:**
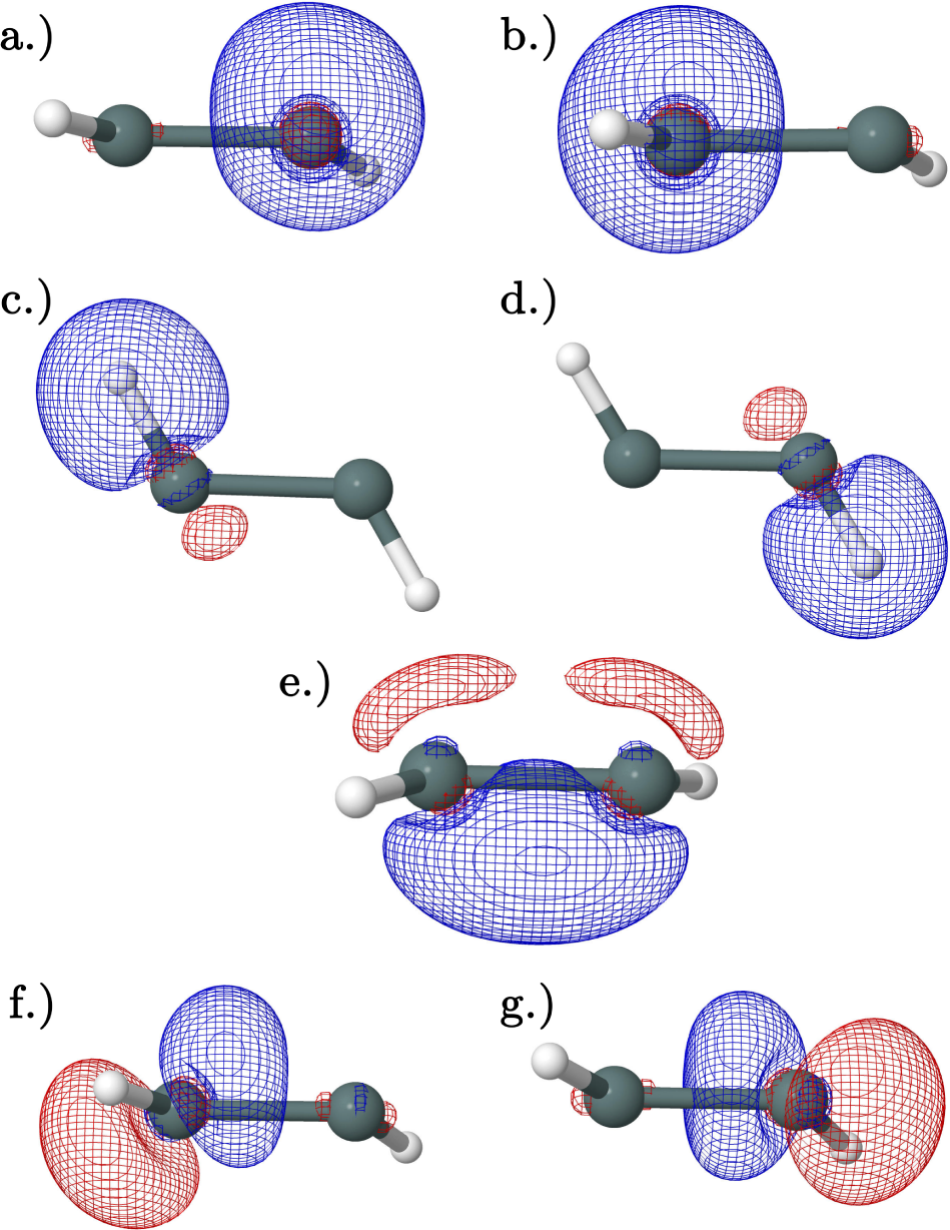
Natural bonding orbitals obtained from NBO analysis of the CCSD(T)/QZ geometry of the neutral nonplanar trans minimum: (a,b) degenerate Sn lone pair orbitals. (c,d) Degenerate Sn–H bonding orbitals. (e) HOMO Sn–Sn bonding orbital with primarily π character. (f) LUMO. (g,h) Important bonding interaction orbitals. Orbitals (a,g) and orbitals (b,h) have a second-order perturbation interaction energy of 93 kcal mol^–1^.

The lack of a planar trans minimum in Sn_2_H_2_ instigated a search for a minimum of a similar geometry: the nonplanar trans minimum (Figure 10). In this structure, there is only one bonding orbital between the Sn atoms (Figure 10e) and there are two Sn–H single bonding orbitals (Figure 10c,d) and two donor–acceptor interactions between a lone pair and an empty π orbital on adjacent Sn atoms with a second-order perturbation energy of 93 kcal mol^–1^: Figure 10a–f and Figure 10b–g. The bonding of the trans minima in this study is more similar to Lein et al.^59^ description of the planar trans structure and is qualitatively similar to the planar trans cation in its orbital construction.

The nonplanar geometry of the trans minima can also be partially explained by the NBO analysis. Figure 10 shows the bonding orbital as well as the four orbitals involved in the donor–acceptor interactions. Figure 10e shows the natural bonding orbital between the Sn atoms; Figure 10a,b shows the lone pairs, while Figure 10f,g shows the lone valence natural bond orbitals. The natural bonding orbital in Figure 10e contains an electron density that is primarily on one side of the molecule. Through Coloumbic repulsion, this seems to shift the lone pair orbitals slightly to the side, breaking the plane of the molecule.

**Figure 11:**
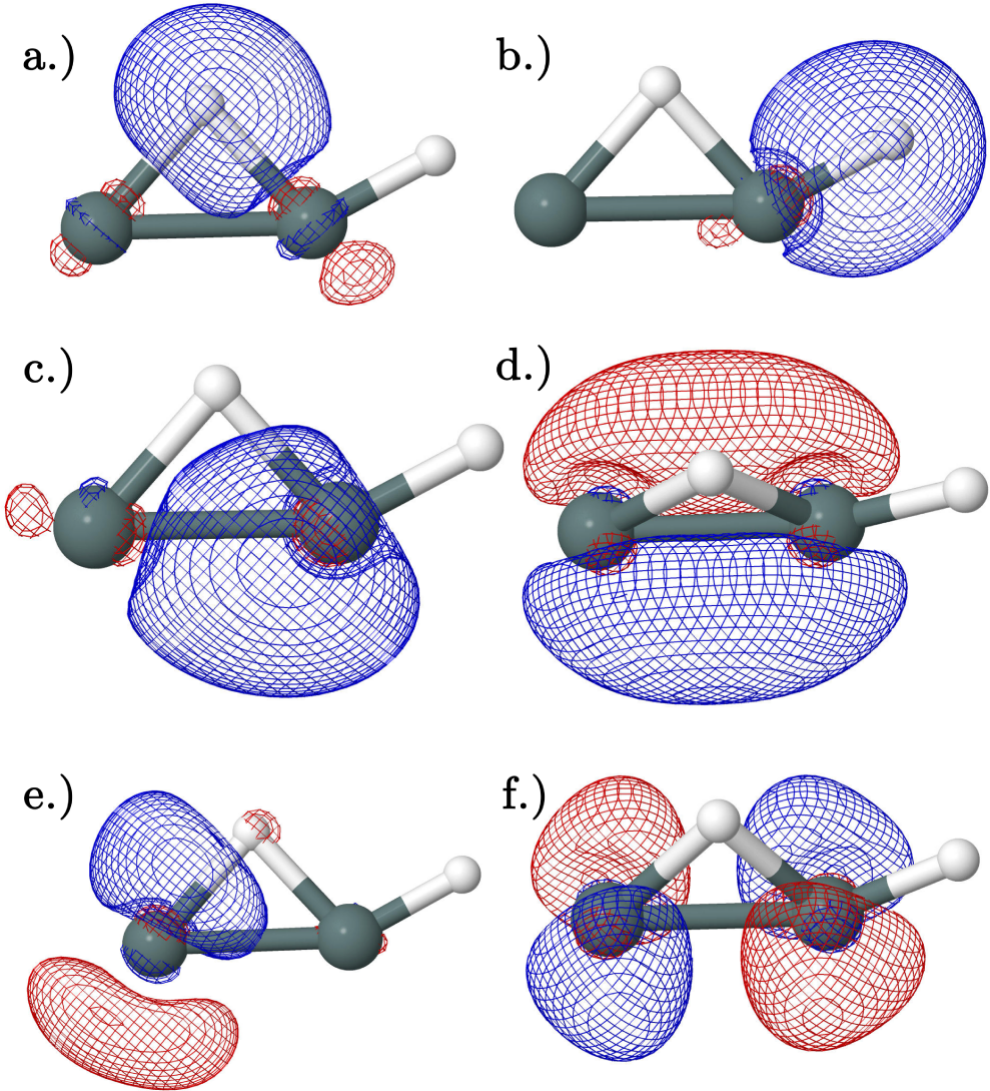
Natural bonding orbitals obtained from NBO analysis of the CCSD(T)/QZ geometry of the neutral and cation monobridged minimum: (a) Sn-bridging H-bonding orbital. (b) Sn-terminal H-bonding orbital. (c) Sn–Sn bonding orbital with the σ character. (d) HOMO/SOMO Sn–Sn bonding orbital with primarily π character. (e) Unoccupied interacting orbital. (f) LUMO. Orbitals (a,e) have second-order perturbation interaction energies of 160 kcal mol^–1^ and 73 kcal mol^–1^ for the neutral and cation, respectively. The cation and neutral are represented in one image as their orbitals are qualitatively similar.

For the monobridged structure, Lein et al.^59^ suggested that there was a donor–acceptor interaction between a lone pair on a Sn atom and a empty valence orbital on the adjacent Sn atom. However, the NBO analysis shows two Sn–H bonds (Figure 11a,b) and two bonding orbitals between the Sn atoms (Figure 11c,d). There is not a lone pair with significant interactions with any other orbitals, but one of the Sn–H bonds interacts with a lone valence orbital in the adjacent Sn atom, leading to the bridging hydrogen: Figure 11a–e. The HOMO (Figure 11f) has no strong interactions.

Further NBO analysis demonstrates that the lost electron between the neutral and cationic structures always originates from the Sn–Sn bond. In each neutral molecule, the HOMO is always a Sn–Sn bonding orbital of either σ or π character, and when molecules lose an electron, the singly occupied molecular orbital is always the same HOMO Sn–Sn bonding orbital. Additionally, for all molecules which contain a donor–acceptor interaction, the second-order perturbation energy of the cation is much smaller than that of the neutral form. The strength of the donor–acceptor interactions decreases as an electron leaves the system, partially destabilizing the molecules.

### Dipole Moments and Partial Charges

**Figure 12:**
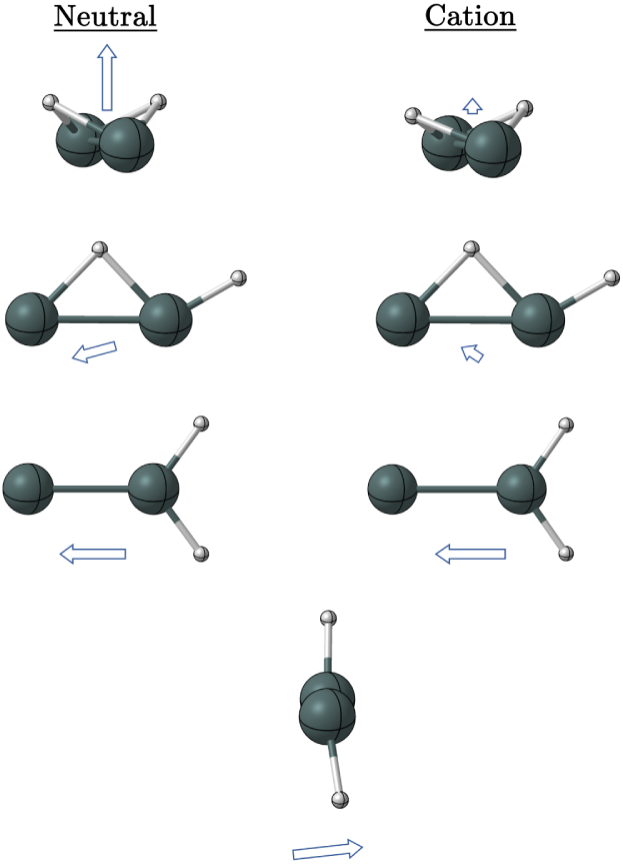
Relative center-of-mass dipole moments for the butterfly neutral, butterfly cation, monobridged neutral, monobridged cation, vinylidene-like neutral, vinylidene-like cation, and nonplanar trans neutral calculated from CCSD(T)/QZ geometries.

Center-of-mass dipole moments and partial charges were calculated for each molecule based on the QZ geometry with the exception of the monobridged transition states, which were only optimized with the TZ basis set. Partial charges, calculated from NBO analysis, are given in Table 13, dipole moments for the minima are presented in Figure 12, and dipole moments of transition states are included in Supporting Information.

**Table 13:** Partial Charges from NBO Analyses of the Butterfly, Monobridged, Vinylidene-Like, Nonplanar Trans, Planar Dibridged, MB-TS1, MB-TS2, MB-TS3, Planar Trans, Perptrans, Cis, and Linear Structures along the Sn_2_H_2_ Potential Energy Surface Calculated from CCSD(T)/QZ Geometries for All Structures except MB-TS1, MB-TS2, and MB-TS3 Which Are Calculated from CCSD(T)/TZ Geometries

structure		neutral	cation	structure		neutral	cation
butterfly	Sn1	+0.36	+0.93	monobridged	Sn1	+0.29	+0.83
	Sn2	+0.36	+0.93		Sn2	+0.24	+0.75
	H1	–0.36	–0.43		H1	–0.30	–0.38
	H2	–0.36	–0.43		H2	–0.22	–0.20
vinylidene-like	Sn1	+0.20	+0.69	nonplanar trans	Sn1	+0.28	
	Sn2	+0.23	+0.69		Sn2	+0.28	
	H1	–0.22	–0.19		H1	–0.28	
	H2	–0.22	–0.19		H2	–0.28	
planar dibridged	Sn1	+0.36	+0.96	MB-TS1	Sn1	+0.45	+0.97
	Sn2	+0.36	+0.96		Sn2	+0.18	+0.72
	H1	–0.36	–0.46		H1	–0.36	–0.43
	H2	–0.36	–0.46		H2	–0.26	–0.27
MB-TS2	Sn1	+0.28	+0.77	MB-TS3	Sn1	+0.21	+0.81
	Sn2	+0.16	+0.66		Sn2	+0.36	+0.73
	H1	–0.25	–0.28		H1	–0.29	–0.28
	H2	–0.19	–0.16		H2	–0.27	–0.25
planar trans	Sn1	+0.27	+0.76	perptrans	Sn1		+0.87
	Sn2	+0.27	+0.76		Sn2		+0.87
	H1	–0.27	–0.26		H1		–0.37
	H2	–0.27	–0.26		H2		–0.37
cis	Sn1	+0.37	+0.89	linear	Sn1	+0.11	+0.57
	Sn2	+0.37	+0.89		Sn2	+0.11	+0.57
	H1	–0.37	–0.39		H1	–0.11	–0.07
	H2	–0.37	–0.39		H2	–0.11	–0.07

Sn atoms are found to be positively partially charged, while the H atoms are negatively partially charged in all neutral and cation structures. As the neutral structures lose an electron, partial charges shift more dramatically on Sn atoms compared to those on H atoms. Partial charges on Sn atoms shift as much as 0.6 in the case of the butterfly minimum, while the partial charge of H atoms changes by 0.1 at most in the case of the planar dibridged transition state. Among the minima, the butterfly structure demonstrates the most dramatic change in the partial charges between the neutral and cation forms.

The dipole moments of the minima reveal that losing an electron from the molecule typically decreases the overall dipole moment (Figure 12). The butterfly’s dipole moment nearly disappears, decreasing from −0.86 to −0.02 D; the monobridged dipole moment decreases from 0.52 to 0.17 D; the vinylidene-like dipole moment increases slightly from 0.84 to 0.91 D. The increases in the dipole moment can also be observed from the partial charges, where the vinylidene-like minimum is the only minimum whose partial charge on both H atoms becomes more positive.

## Conclusions

The geometries, relative energies, vibrational frequencies, and bond orders have been presented for many structures of Sn_2_H_2_ and Sn_2_H_2_^+^ in this study. The geometries for the butterfly, monobridged, vinylidene-like, nonplanar trans, planar dibridged, perptrans, planar trans, cis, and linear were found using the CCSD(T) method with a cc-pwCVQZ-PP basis set having a 28-electron effective core potential. Additionally, three monobridged-like transition states have been identified using the CCSD(T) method with a cc-pwCVTZ-PP basis set. The butterfly isomer lies lowest in energy on the potential energy surfaces of both the neutral and cation species of Sn_2_H_2_. Energy minima stationary points present in other group 14 metal hydrides, M_2_H_2_ (M = Si, Ge, Sn, and Pb) were confirmed, such as the monobridged, vinylidene-like, and planar trans isomers, but the planar trans isomer was identified to be a transition state disagreeing with former research. Instead, through rigorous coupled cluster calculations, a newly identified nonplanar trans minimum was determined to lie along the potential energy surface, revealing a PES of Sn_2_H_2_ differing from the one provided by Nagase et al.^25^ Focal point analysis provided energies approaching the FCI and CBS with a rapid convergence. Harmonic vibrational frequencies were also obtained with the CCSD(T) method with a cc-pwVCTZ-PP basis set. Our neutral species predictions were compared to the experimental assignments of Wang and co-workers,^28^ and some discrepancies were found.

Furthermore, the neutral and cation structures were examined closely to identify differences between their electronic structures and to provide theoretical data to assist in the detection of the cation. The primary geometric differences were related to the Sn–Sn bonds, where bond lengths and bond orders changed the most in geometry and electron occupation. The bond lengths typically increased in the cation, and bond orders decreased by roughly 0.5 among all structures. The changing geometries and differences in electronic occupation indicate that the lost electron in the Sn_2_H_2_ cation originates from the Sn–Sn bond. Additionally, harmonic vibrational analysis of the cation revealed vibrational modes and intensities, which may assist in the laboratory detection of the cation. Generally, our analysis of the neutral and cation Sn_2_H_2_ highlights the importance of studying the electronic structure with rigorous, high-level theory.

## Supporting Information Available

Cartesian coordinates, numeric center-of-mass dipole moments, focal point approach tables, and natural bond orbitals (PDF)
