# Supplementary material for: Comparative Study of Neutral and Cationic Sn2H2: Toward Laboratory Detection of the Cation
Source: J Phys Chem A. 2024 Aug 19;128(34):7090–104. doi: 10.1021/acs.jpca.4c03220 (PMC11372748; doi:10.1021/acs.jpca.4c03220)
Supplement: Supplementary file 1 — jp4c03220_si_001.pdf [file jp4c03220_si_001.pdf]

Supporting Information: Comparative Neutral and Cationic Sn_2H_2: Towards Laboratory Detection of the Cation

Samuel Biggerstaff[†], Nathaniel L. Kitzmiller[†], Justin M. Turney[†], and Henry F. Schaefer III[†*]

†Center for Computational Quantum Chemistry

Department of Chemistry

University of Georgia

Athens, GA 30602, United States

Email: ccq@uga.edu

Contents

S1 Overview S1

S2 Geometries S2

S2.1 Minima . . . S2

S2.2 Transition States . . . S3

S2.3 MB-TS . . . S4

S3 Center of Mass Dipole Moments S5

S4 Focal Point Analysis S6

S4.1 Monobridged . . . S6

S4.2 Vinylidene-like . . . S7

S4.3 Nonplanar Trans . . . S8

S4.4 Planar Dibridged . . . S8

S4.5 Planar Trans . . . S9

S4.6 Perptrans . . . S10

S4.7 Cis . . . S11

S4.8 Linear . . . S12

S5 Natural Bond Orbitals S13

S5.1 Butterfly . . . S13

S5.2 Monobridged . . . S15

S5.3 Vinylidene-like . . . S17

S5.4 Nonplanar Trans . . . S19

S5.5 Planar Dibridged . . . S20

S5.6 Planar Trans . . . S22

S5.7 Perptrans . . . S24

S5.8 Cis . . . S25

S5.9 Linear . . . S27

S1 Overview

Cartesian coordinates in Bohr for minima, transition states, and MB-TS structures are provided in section S2, center of mass dipole moments in Debeye relative to the corresponding structures Cartesian coordinates for all structures are provided in section S3, focal point approach tables for all structures except the monobridged transition state structures are provided in section S4, and images for relevant natural bond orbitals, including, HOMOs, SOMOs, LUMOs, bonding orbitals, and donor-acceptor interacting orbitals, obtained from NBO analysis are provided in section S5.

S2 Geometries

S2.1 Minima

Table S1: Cartesian Coordinates in Bohr for the butterfly, monobridged, vinylidene-like, and nonplanar trans minima calculated at the CCSD(T)/QZ level of theory.

Molecule	Form	Atom	X	Y	Z
Butterfly	Neutral	Sn1	0.000000000000	1.363051985283	0.006652033836
		Sn2	0.000000000000	−1.363051985283	0.006652033836
		H1	1.127574253457	0.000000000000	−0.791400765714
		H2	−1.127574253457	0.000000000000	−0.791400765714
	Cation	Sn1	0.000000000000	1.439384982346	0.005488814879
		Sn2	0.000000000000	−1.439384982346	0.005488814879
		H1	1.129156894451	0.000000000000	−0.653011154992
		H2	−1.129156894451	0.000000000000	−0.653011154992
Monobridged	Neutral	Sn1	−0.956201170443	−0.885948535400	0.000000000000
		Sn2	0.951182932144	0.856249776168	0.000000000000
		H1	−0.956201170443	1.078217838194	0.000000000000
		H2	1.553227242539	2.455080605137	0.000000000000
	Cation	Sn1	−0.978530191185	−0.951893497462	0.000000000000
		Sn2	0.974591715090	0.921828369047	0.000000000000
		H1	−0.978530191185	1.018420212162	0.000000000000
		H2	1.447095606591	2.558465630178	0.000000000000
Vinylidene-like	Neutral	Sn1	0.000000000000	0.000000000000	−1.339566202488
		Sn2	0.000000000000	0.000000000000	1.300811455995
		H1	0.000000000000	1.370415274062	2.305350273887
		H2	0.000000000000	−1.370415274062	2.305350273887
	Cation	Sn1	0.000000000000	0.000000000000	−1.414990115783
		Sn2	0.000000000000	0.000000000000	1.375644666574
		H1	0.000000000000	1.389727257275	2.340488593510
		H2	0.000000000000	−1.389727257275	2.340488593510
Nonplanar Trans	Neutral	Sn1	0.020988529022	1.315805323419	0.001098246505
		Sn2	−0.020988529078	−1.315805323581	0.001098246505
		H1	−1.449527988178	2.243502133519	−0.130659757395
		H2	1.449527994822	−2.243502114181	−0.130659756795

S2.2 Transition States

Table S2: Cartesian Coordinates in Bohr for the planar dibridged, planar trans, perptrans, cis, and linear transition states calculated at the CCSD(T)/QZ level of theory.

Molecule	Form	Atom	X	Y	Z
Planar Dibridged	Neutral	Sn1	0.000000000000	1.449053626924	0.000000000000
		Sn2	0.000000000000	−1.449053626924	0.000000000000
		H1	1.195161172922	0.000000000000	0.000000000000
		H2	−1.195161172922	0.000000000000	0.000000000000
	Cation	Sn1	0.000000000000	1.522468635125	0.000000000000
		Sn2	0.000000000000	−1.522468635125	0.000000000000
		H1	1.161611889910	0.000000000000	0.000000000000
		H2	−1.161611889910	0.000000000000	0.000000000000
Planar Trans	Neutral	Sn1	1.070978878828	−0.697964481566	0.000000000000
		Sn2	−1.070978878828	0.697964481566	0.000000000000
		H1	1.070978878828	−2.414403281105	0.000000000000
		H2	−1.070978878828	2.414403281105	0.000000000000
	Cation	Sn1	1.142749199179	−0.711603595920	0.000000000000
		Sn2	−1.142749199179	0.711603595920	0.000000000000
		H1	1.142749199179	−2.425671921775	0.000000000000
		H2	−1.142749199179	2.425671921775	0.000000000000
Perptrans	Cation	Sn1	1.534029461852	−0.231506546599	0.000000000000
		Sn2	−1.534029461852	0.231506546599	0.000000000000
		H1	1.534029461852	−1.972062326507	0.000000000000
		H2	−1.534029461852	1.972062326507	0.000000000000
Cis	Neutral	Sn1	0.000000000000	1.514133688482	0.014631788793
		Sn2	0.000000000000	−1.514133688482	0.014631788793
		H1	0.000000000000	1.612094004776	−1.740762169992
		H2	0.000000000000	−1.612094004776	−1.740762169992
	Cation	Sn1	0.000000000000	1.617285112858	0.014497625352
		Sn2	0.000000000000	−1.617285112858	0.014497625352
		H1	0.000000000000	1.783531616642	−1.724800578005
		H2	0.000000000000	−1.783531616642	−1.724800578005
Linear	Neutral	Sn1	0.000000000000	1.184520969200	0.000000000000
		Sn2	0.000000000000	−1.184520969200	0.000000000000
		H1	0.000000000000	2.829199947835	0.000000000000
		H2	0.000000000000	−1.184520969200	0.000000000000
	Cation	Sn1	0.000000000000	1.213210154595	0.000000000000
		Sn2	0.000000000000	−1.213210154595	0.000000000000
		H1	0.000000000000	2.864446413045	0.000000000000
		H2	0.000000000000	−2.864446413045	0.000000000000

S2.3 MB-TS

Table S3: Cartesian Coordinates in Bohr for the MB-TS1, MB-TS2, and MB-TS3 transition states calculated at the CCSD(T)/TZ level of theory.

Molecule	Form	Atom	X	Y	Z
MB-1	Neutral	Sn1	1.3552830757	0.0013107932	0.0058366504
		Sn2	−1.3389551635	0.0188515585	−0.0030846377
		H1	0.0511227557	−0.9735239347	−1.0197702004
		H2	−1.9936744760	−1.4252162728	0.6923599707
	Cation	Sn1	1.4229249776	0.0017059457	0.0055864395
		Sn2	−1.4076147103	0.0185657075	−0.0020474027
		H1	0.1061831245	−0.8991384678	−1.0326300542
		H2	−1.9276652552	−1.5126055297	0.6115863818
MB-2	Neutral	Sn1	−1.3159834786	−0.0030944328	0.0000000000
		Sn2	1.2825129117	−0.0086625416	0.0000000000
		H1	1.0404758001	1.7238637240	0.0000000000
		H2	2.9415591327	−0.3251223583	0.0000000000
	Cation	Sn1	−1.3715045280	−0.0031829800	0.0000000000
		Sn2	1.3386029431	−0.0096325023	0.0000000000
		H1	0.8990939217	1.6929809556	0.0000000000
		H2	3.0152486098	−0.1683072001	0.0000000000
MB-3	Neutral	Sn1	−1.3075923784	−0.0177775139	0.0000000000
		Sn2	1.2942369660	0.0085996965	0.0000000000
		H1	−1.2947848391	1.7323111868	0.0000000000
		H2	−1.3075923784	−0.0177775139	0.0000000000
	Cation	Sn1	1.3536232213	0.0183890417	0.0000000000
		Sn2	−1.3573136664	−0.0208443183	0.0000000000
		H1	2.4899616285	−1.2614981507	0.0000000000
		H2	−2.0509049796	1.5536056961	0.0000000000

S3 Center of Mass Dipole Moments

Table S4: Relative center of mass dipole moments in Debye for the neutral and cationic butterfly, monobridged, vinylidene-like, nonplanar trans, planar dibridged, MB-TS1, MB-TS2, MB-TS3, planar trans, perptrans, cis, and linear structures calculated from CCSD(T)/QZ geometries except for the MB-TS structures which were calculated from CCSD(T)/TZ geometries.

Molecule	Form	X	Y	Z
Butterfly	Neutral	0.00	0.00	−0.86
	Cation	0.00	0.00	−0.02
Monobridged	Neutral	−0.33	−0.40	0.00
	Cation	−0.16	−0.06	0.00
Vinylidene-like	Neutral	0.00	0.00	−0.84
	Cation	0.00	0.00	−0.91
Nonplanar Trans	Neutral	0.00	0.00	−0.94
Planar Dibridged	Neutral	0.00	0.00	0.00
	Cation	0.00	0.00	0.00
MB-1	Neutral	0.98	0.20	0.81
	Cation	1.11	0.09	0.12
MB-2	Neutral	−1.04	−0.86	0.00
	Cation	−0.77	−0.59	0.00
MB-3	Neutral	0.72	−0.23	0.00
	Cation	0.28	−0.01	0.00
Planar Trans	Neutral	0.00	0.00	0.00
	Cation	0.00	0.00	0.00
Perptrans	Cation	0.00	0.00	0.00
Cis	Neutral	0.00	0.00	−0.76
	Cation	0.00	0.00	−0.33
Linear	Neutral	0.00	0.00	0.00
	Cation	0.00	0.00	0.00

S4 Focal Point Analysis

S4.1 Monobridged

S4.1.1 Neutral

Table S5: Focal point analysis table for the neutral monobridged isomer relative to the neutral butterfly isomer in kcal mol^{-1}. HF energies are extrapolated to the CBS Limit using a three-point extrapolation formula, and post HF energies are extrapolated using a two-point extrapolation method. Corrections for CCSDT, CCSDT(Q), and the zero point vibration energy (ZPVE) are added to obtain the CCSDT(Q)/CBS energy.

Monobridged Neutral	HF	+δMP2	+δCCSD	+δ(T)	Net
DZ	17.31	−3.88	+1.35	−1.63	[13.16]
TZ	17.18	−3.55	+1.25	−1.65	[13.23]
QZ	17.19	−3.56	+1.30	−1.70	[13.23]
5Z	17.19	−3.55	+1.34	−1.71	[13.27]
CBS Limit	[17.19]	[−3.56]	[+1.32]	[−1.73]	[**13.23**]

$$\text{CCSD(T)/CBS} + \Delta\text{ZPVE} + \Delta\text{T} + \Delta\text{Q} = \text{CCSDT(Q)/CBS} = \mathbf{13.23} - 1.44 + 0.12 - 0.12 = 11.79$$

S4.1.2 Cation

Table S6: Focal point analysis table for the cation monobridged isomer relative to the cation butterfly isomer in kcal mol^{-1}. HF energies are extrapolated to the CBS Limit using a three-point extrapolation formula, and post HF energies are extrapolated using a two-point extrapolation method. Corrections for CCSDT, CCSDT(Q), and the zero point vibration energy (ZPVE) are added to obtain the CCSDT(Q)/CBS energy.

Monobridged Cation	HF	+δMP2	+δCCSD	+δ(T)	Net
DZ	14.16	+1.97	−0.25	−0.69	[15.19]
TZ	14.58	+1.89	−0.78	−0.62	[15.07]
QZ	14.59	+1.74	−0.81	−0.63	[14.89]
5Z	14.60	+1.66	−0.77	−0.64	[14.85]
CBS Limit	[14.60]	[+1.66]	[−0.83]	[−0.64]	[**14.78**]

$$\text{CCSD(T)/CBS} + \Delta\text{ZPVE} + \Delta\text{T} + \Delta\text{Q} = \text{CCSDT(Q)/CBS} = \mathbf{14.78} - 0.89 - 0.04 - 0.01 = 13.84$$

S4.2 Vinylidene-like

S4.2.1 Neutral

Table S7: Focal point analysis table for the neutral vinylidene-like isomer relative to the neutral butterfly isomer in kcal mol^{-1}. HF energies are extrapolated to the CBS Limit using a three-point extrapolation formula, and post HF energies are extrapolated using a two-point extrapolation method. Corrections for CCSDT, CCSDT(Q), and the zero point vibration energy (ZPVE) are added to obtain the CCSDT(Q)/CBS energy.

Vinylidene-like Neutral	HF	$+\delta$MP2	$+\delta$CCSD	$+\delta$(T)	Net
DZ	10.12	+9.24	−3.75	−0.05	[15.57]
TZ	10.85	+11.07	−5.15	+0.37	[17.14]
QZ	10.87	+11.35	−5.34	+0.48	[17.35]
5Z	10.87	+11.36	−5.31	+0.50	[17.43]
CBS Limit	[10.87]	[+11.51]	[−5.45]	[+0.54]	[**17.46**]

$$\text{CCSD(T)/CBS} + \Delta\text{ZPVE} + \Delta\text{T} + \Delta\text{Q} = \text{CCSDT(Q)/CBS} = \mathbf{17.46} - 0.40 - 0.10 - 0.04 = 16.92$$

S4.2.2 Cation

Table S8: Focal point analysis table for the cation vinylidene-like isomer relative to the cation butterfly isomer in kcal mol^{-1}. HF energies are extrapolated to the CBS Limit using a three-point extrapolation formula, and post HF energies are extrapolated using a two-point extrapolation method. Corrections for CCSDT, CCSDT(Q), and the zero point vibration energy (ZPVE) are added to obtain the CCSDT(Q)/CBS energy.

Vinylidene-like Cation	HF	$+\delta$MP2	$+\delta$CCSD	$+\delta$(T)	Net
DZ	3.67	+13.50	−3.24	+1.05	[14.99]
TZ	5.22	+14.58	−5.03	+1.52	[16.29]
QZ	5.29	+14.68	−5.30	+1.64	[16.31]
5Z	5.30	+14.60	−5.28	+1.67	[16.30]
CBS Limit	[5.30]	[+14.75]	[−5.46]	[+1.71]	[**16.30**]

$$\text{CCSD(T)/CBS} + \Delta\text{ZPVE} + \Delta\text{T} + \Delta\text{Q} = \text{CCSDT(Q)/CBS} = \mathbf{16.30} - 0.08 + 0.16 - 0.13 = 16.25$$

S4.3 Nonplanar Trans

S4.3.1 Neutral

Table S9: Focal point analysis table for the neutral planar trans structure relative to the neutral butterfly isomer in kcal mol^{-1}. HF energies are extrapolated to the CBS Limit using a three-point extrapolation formula, and post HF energies are extrapolated using a two-point extrapolation method. Corrections for CCSDT, CCSDT(Q), and the zero point vibration energy (ZPVE) are added to obtain the CCSDT(Q)/CBS energy.

Nonplanar Trans Neutral	HF	$+\delta$MP2	$+\delta$CCSD	$+\delta$(T)	Net
DZ	26.87	+1.08	−2.44	−2.87	[22.63]
TZ	27.36	+2.52	−2.80	−2.66	[24.42]
QZ	27.41	+2.86	−2.77	−2.68	[24.83]
5Z	24.43	+2.91	−2.69	−2.69	[24.96]
CBS Limit	[27.43]	[+3.08]	[−2.74]	[−2.69]	[**25.11**]

$$\mathrm{CCSD(T)/CBS} + \Delta\mathrm{ZPVE} + \Delta\mathrm{T} + \Delta\mathrm{Q} = \mathrm{CCSDT(Q)/CBS} = \mathbf{25.11} - 1.25 - 0.11 + 0.81 = 24.56$$

S4.4 Planar Dibridged

S4.4.1 Neutral

Table S10: Focal point analysis table for the neutral planar dibridged structure relative to the neutral butterfly isomer in kcal mol^{-1}. HF energies are extrapolated to the CBS Limit using a three-point extrapolation formula, and post HF energies are extrapolated using a two-point extrapolation method. Corrections for CCSDT, CCSDT(Q), and the zero point vibration energy (ZPVE) are added to obtain the CCSDT(Q)/CBS energy.

Planar Neutral	HF	$+\delta$MP2	$+\delta$CCSD	$+\delta$(T)	Net
DZ	10.70	−6.23	+1.25	−1.60	[4.12]
TZ	10.28	−5.19	+1.77	−1.68	[5.19]
QZ	10.18	−4.84	+1.90	−1.67	[5.57]
5Z	10.18	−4.66	+1.93	−1.67	[5.78]
CBS Limit	[10.18]	[−4.64]	[+1.97]	[−1.67]	[**5.84**]

$$\mathrm{CCSD(T)/CBS} + \Delta\mathrm{ZPVE} + \Delta\mathrm{T} + \Delta\mathrm{Q} = \mathrm{CCSDT(Q)/CBS} = \mathbf{5.84} + 0.07 + 0.10 - 0.18 = 5.83$$

S4.4.2 Cation

Table S11: Focal point analysis table for the cation planar dibridged structure relative to the cation butterfly isomer in kcal mol^{-1}. HF energies are extrapolated to the CBS Limit using a three-point extrapolation formula, and post HF energies are extrapolated using a two-point extrapolation method. Corrections for CCSDT, CCSDT(Q), and the zero point vibration energy (ZPVE) are added to obtain the CCSDT(Q)/CBS energy.

Planar Dibridged Cation	HF	$+\delta$MP2	$+\delta$CCSD	$+\delta$(T)	Net
DZ	1.39	−0.47	+0.33	−0.22	[1.02]
TZ	1.67	−0.09	+0.36	−0.23	[1.71]
QZ	1.68	−0.01	+0.39	−0.22	[1.86]
5Z	1.68	−0.08	+0.39	−0.21	[1.94]
CBS Limit	[1.69]	[−0.07]	[+0.40]	[−0.21]	[**1.95**]

$$\text{CCSD(T)/CBS} + \Delta\text{ZPVE} + \Delta\text{T} + \Delta\text{Q} = \text{CCSDT(Q)/CBS} = \mathbf{1.95} - 0.26 - 0.01 - 0.02 = 1.66$$

S4.5 Planar Trans

S4.5.1 Neutral

Table S12: Focal point analysis table for the neutral planar trans structure relative to the neutral butterfly isomer in kcal mol^{-1}. HF energies are extrapolated to the CBS Limit using a three-point extrapolation formula, and post HF energies are extrapolated using a two-point extrapolation method. Corrections for CCSDT, CCSDT(Q), and the zero point vibration energy (ZPVE) are added to obtain the CCSDT(Q)/CBS energy.

Planar Trans Neutral	HF	$+\delta$MP2	$+\delta$CCSD	$+\delta$(T)	Net
DZ	28.03	−3.43	+1.05	−2.52	[23.13]
TZ	28.31	−2.23	+0.49	−2.35	[24.23]
QZ	28.36	−2.06	+0.52	−2.37	[24.45]
5Z	28.37	−2.02	+0.59	−2.38	[24.56]
CBS Limit	[28.38]	[−1.93]	[+0.54]	[−2.38]	[**24.60**]

$$\text{CCSD(T)/CBS} + \Delta\text{ZPVE} + \Delta\text{T} + \Delta\text{Q} = \text{CCSDT(Q)/CBS} = \mathbf{24.60} - 1.30 + 0.02 - 0.51 = 22.82$$

S4.5.2 Cation

Table S13: Focal point analysis table for the cation planar trans structure relative to the cation butterfly isomer in kcal mol^{-1}. HF energies are extrapolated to the CBS Limit using a three-point extrapolation formula, and post HF energies are extrapolated using a two-point extrapolation method. Corrections for CCSDT, CCSDT(Q), and the zero point vibration energy (ZPVE) are added to obtain the CCSDT(Q)/CBS energy.

Planar Trans Cation	HF	$+\delta$MP2	$+\delta$CCSD	$+\delta$(T)	Net
DZ	24.39	+4.27	−1.18	−1.28	[26.20]
TZ	25.53	+4.98	−2.28	−1.05	[27.18]
QZ	25.51	+5.05	−2.36	−1.03	[27.18]
5Z	25.52	+5.00	−2.29	−1.03	[27.20]
CBS Limit	[25.52]	[+5.10]	[−2.41]	[−1.00]	[**27.20**]

$$\text{CCSD(T)/CBS} + \Delta\text{ZPVE} + \Delta\text{T} + \Delta\text{Q} = \text{CCSDT(Q)/CBS} = \mathbf{27.20} - 1.39 - 0.17 - 0.26 = 25.38$$

S4.6 Perptrans

S4.6.1 Cation

Table S14: Focal point analysis table for the cation trans structure with bond angles of nearly 90° (pertrans) relative to the cation butterfly isomer in kcal mol^{-1}. HF energies are extrapolated to the CBS Limit using a three-point extrapolation formula, and post HF energies are extrapolated using a two-point extrapolation method. Corrections for CCSDT, CCSDT(Q), and the zero point vibration energy (ZPVE) are added to obtain the CCSDT(Q)/CBS energy.

Perptrans Cation	HF	$+\delta$MP2	$+\delta$CCSD	$+\delta$(T)	Net
DZ	13.21	+11.94	−3.72	+1.89	[23.31]
TZ	15.16	+13.44	−5.12	+2.40	[25.89]
QZ	15.29	+13.81	−5.25	+2.56	[26.40]
5Z	15.31	+13.82	−5.20	+2.60	[26.53]
CBS Limit	[15.31]	[+14.02]	[−5.33]	[+2.64]	[**26.65**]

$$\text{CCSD(T)/CBS} + \Delta\text{ZPVE} + \Delta\text{T} + \Delta\text{Q} = \text{CCSDT(Q)/CBS} = \mathbf{26.65} - 1.39 + 0.06 - 0.00 = 25.32$$

S4.7 Cis

S4.7.1 Neutral

Table S15: Focal point analysis table for the neutral cis isomer relative to the neutral butterfly isomer in kcal mol^{-1}. HF energies are extrapolated to the CBS Limit using a three-point extrapolation formula, and post HF energies are extrapolated using a two-point extrapolation method. Corrections for CCSDT, CCSDT(Q), and the zero point vibration energy (ZPVE) are added to obtain the CCSDT(Q)/CBS energy.

Cis Neutral	HF	$+\delta$MP2	$+\delta$CCSD	$+\delta$(T)	Net
DZ	13.48	+17.59	−6.70	+2.47	[26.84]
TZ	14.98	+19.75	−8.45	+3.09	[29.37]
QZ	15.06	+20.30	−8.67	+3.25	[29.94]
5Z	15.05	+20.34	−8.64	+3.29	[30.05]
CBS Limit	[15.05]	[+20.62]	[−8.80]	[+3.35]	[**30.22**]

$$\text{CCSD(T)/CBS} + \Delta\text{ZPVE} + \Delta\text{T} + \Delta\text{Q} = \text{CCSDT(Q)/CBS} = \mathbf{30.22} - 1.34 - 0.29 - 0.03 = 28.56$$

S4.7.2 Cation

Table S16: Focal point analysis table for the cation cis isomer relative to the cation butterfly isomer in kcal mol^{-1}. HF energies are extrapolated to the CBS Limit using a three-point extrapolation formula, and post HF energies are extrapolated using a two-point extrapolation method. Corrections for CCSDT, CCSDT(Q), and the zero point vibration energy (ZPVE) are added to obtain the CCSDT(Q)/CBS energy.

Cis Cation	HF	$+\delta$MP2	$+\delta$CCSD	$+\delta$(T)	Net
DZ	15.19	+12.67	−4.22	+2.08	[25.72]
TZ	16.87	+14.54	−5.49	+2.60	[28.53]
QZ	16.98	+14.99	−5.59	+2.77	[29.14]
5Z	16.99	+15.06	−5.54	+2.82	[29.32]
CBS Limit	[17.00]	[+15.25]	[−5.66]	[+2.86]	[**29.45**]

$$\text{CCSD(T)/CBS} + \Delta\text{ZPVE} + \Delta\text{T} + \Delta\text{Q} = \text{CCSDT(Q)/CBS} = \mathbf{29.45} - 1.97 + 0.06 - 0.01 = 27.53$$

S4.8 Linear

S4.8.1 Neutral

Table S17: Focal point analysis table for the neutral linear isomer relative to the neutral butterfly isomer in kcal mol^{-1}. HF energies are extrapolated to the CBS Limit using a three-point extrapolation formula, and post HF energies are extrapolated using a two-point extrapolation method. Corrections for CCSDT, CCSDT(Q), and the zero point vibration energy (ZPVE) are added to obtain the CCSDT(Q)/CBS energy.

Linear Neutral	HF	$+\delta$MP2	$+\delta$CCSD	$+\delta$(T)	Net
DZ	65.13	−1.80	+1.33	−3.06	[61.60]
TZ	64.44	−1.59	+0.34	−3.11	[60.07]
QZ	64.45	−1.92	+0.17	−3.16	[59.54]
5Z	64.45	−2.18	+0.23	−3.17	[59.33]
CBS Limit	[64.45]	[−2.10]	[+0.07]	[−3.19]	[**59.22**]

$$\text{CCSD(T)/CBS} + \Delta\text{ZPVE} + \Delta\text{T} + \Delta\text{Q} = \text{CCSDT(Q)/CBS} = \mathbf{59.22} - 0.52 + 0.27 - 0.23 = 58.74$$

S4.8.2 Cation

Table S18: Focal point analysis table for the cation linear structure relative to the cation butterfly isomer in kcal mol^{-1}. HF energies are extrapolated to the CBS Limit using a three-point extrapolation formula, and post HF energies are extrapolated using a two-point extrapolation method. Corrections for CCSDT, CCSDT(Q), and the zero point vibration energy (ZPVE) are added to obtain the CCSDT(Q)/CBS energy.

Linear Cation	HF	$+\delta$MP2	$+\delta$CCSD	$+\delta$(T)	Net
DZ	69.73	+6.46	−1.62	−2.26	[72.31]
TZ	69.60	+5.99	−3.41	−2.18	[70.00]
QZ	69.40	+5.40	−3.66	−2.19	[68.94]
5Z	69.38	+5.06	−3.59	−2.20	[68.66]
CBS Limit	[69.38]	[+5.06]	[−3.81]	[−2.20]	[**68.43**]

$$\text{CCSD(T)/CBS} + \Delta\text{ZPVE} + \Delta\text{T} + \Delta\text{Q} = \text{CCSDT(Q)/CBS} = \mathbf{68.43} - 0.58 + 0.15 - 0.09 = 67.91$$

S4 Natural Bond Orbitals

S5.1 Butterfly

S5.1.1 Neutral

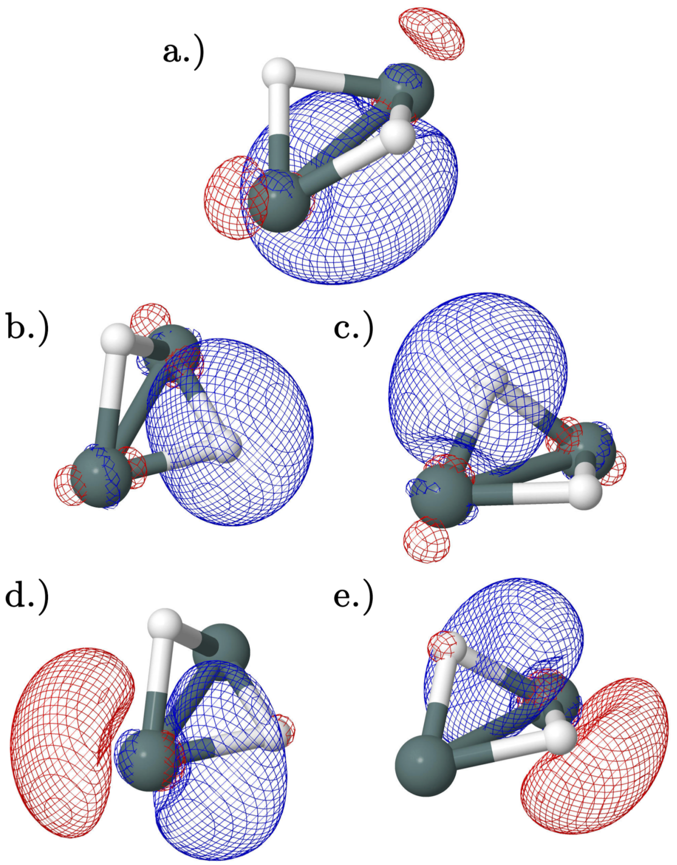

Figure S1: Natural bonding orbitals obtained from NBO analysis of CCSD(T)/QZ geometries of the neutral butterfly minimum: a.) Sn-Sn bonding orbital with primarily π character. (HOMO) b-c.) Degenerate Sn-H bonding orbitals d-e.) Degenerate lowest unoccupied molecular orbitals (LUMO) Orbitals b and d as well as orbitals c and e have a second order perturbation interaction energy of 160.57 kcal mol^{-1}.

S5.1.2 Cation

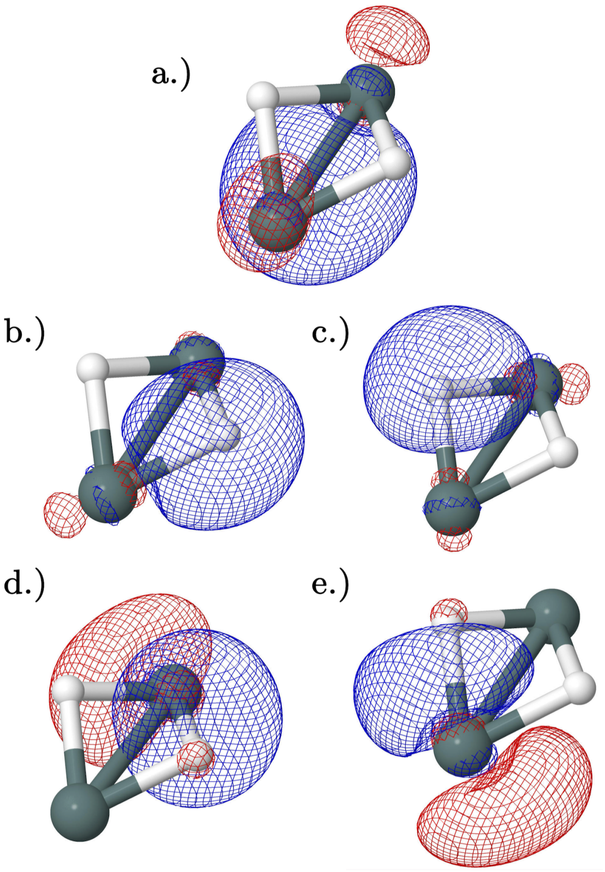

Figure S2: Natural bonding orbitals obtained from NBO analysis of CCSD(T)/QZ geometries of the cation butterfly minimum: a.) Sn-Sn bonding orbital with primarily π character. (SOMO) b-c.) Degenerate Sn-H bonding orbitals d-e.) Degenerate lowest unoccupied molecular orbitals (LUMO) Orbitals b and d as well as orbitals c and e have a second order perturbation interaction energy of 67.47 kcal mol^{-1}.

S5.2 Monobridged

S5.2.1 Neutral

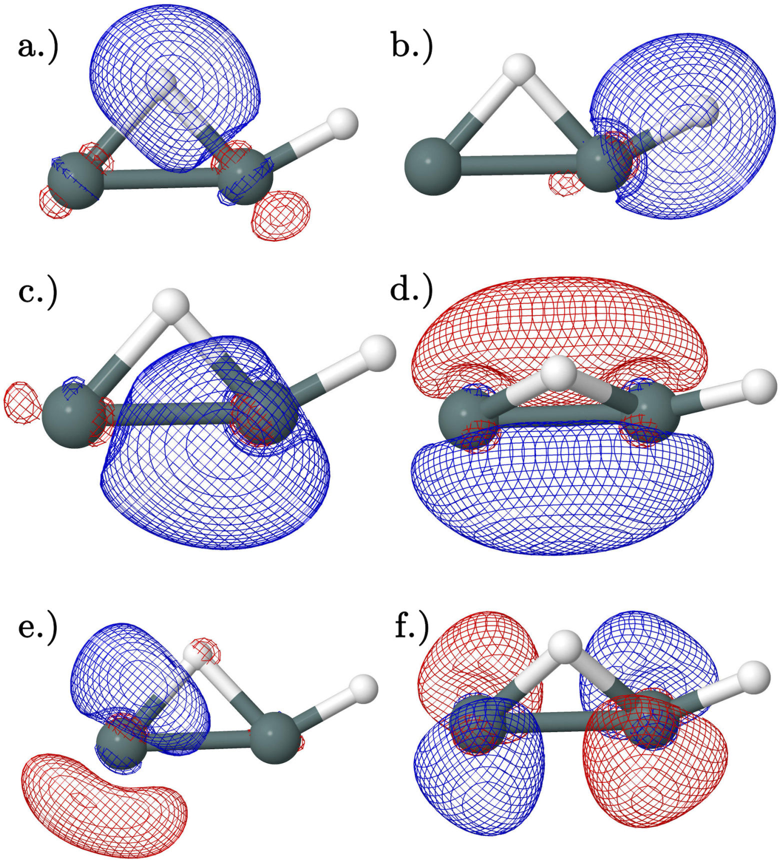

Figure S3: Natural bonding orbitals obtained from NBO analysis of CCSD(T)/QZ geometry of the neutral monobridged minimum: a.) Sn-Bridging H bonding orbital. b.) Sn-Terminal H bonding orbital. c.) Sn-Sn bonding orbital with σ character. d.) HOMO Sn-Sn bonding orbital with primarily π character. e.) Unoccupied interacting orbital f.) LUMO. Orbitals a and e have a second order perturbation interaction energy of 159.85 kcal mol^{-1}.

S5.2.2 Cation

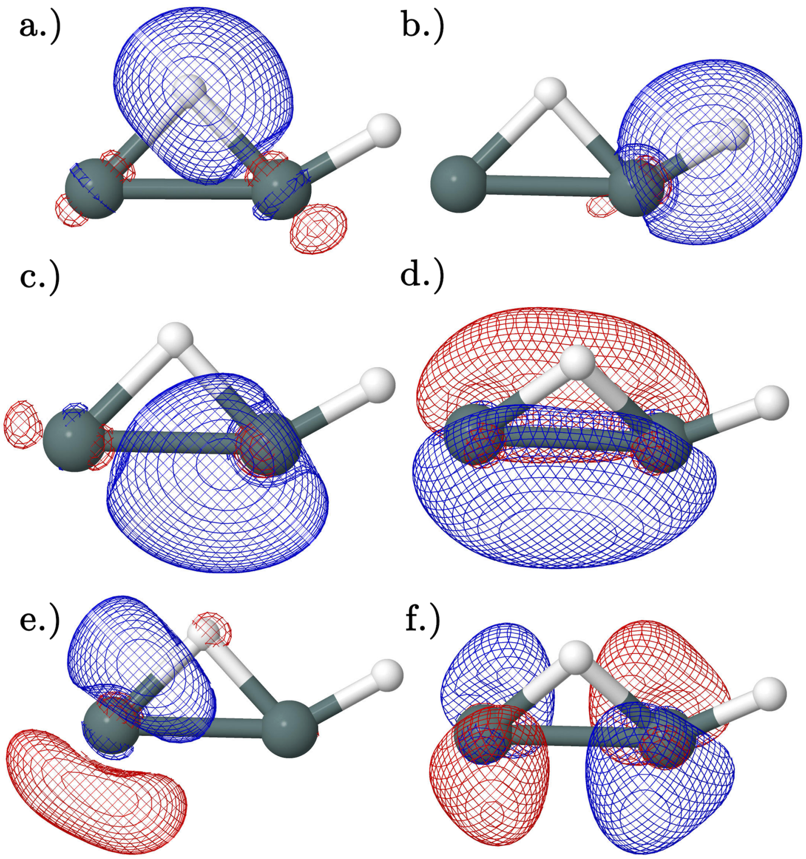

Figure S4: Natural bonding orbitals obtained from NBO analysis of CCSD(T)/QZ geometry of the cation mono-bridged minimum: a.) Sn-Bridging H bonding orbital. b.) Sn-Terminal H bonding orbital. c.) Sn-Sn bonding orbital with σ character. d.) SOMO Sn-Sn bonding orbital with primarily π character. e.) Unoccupied interacting orbital f.) LUMO. Orbitals a and e have a second order perturbation interaction energy of 72.84 kcal mol^{-1}.

S5.3 Vinylidene-like

S5.3.1 Neutral

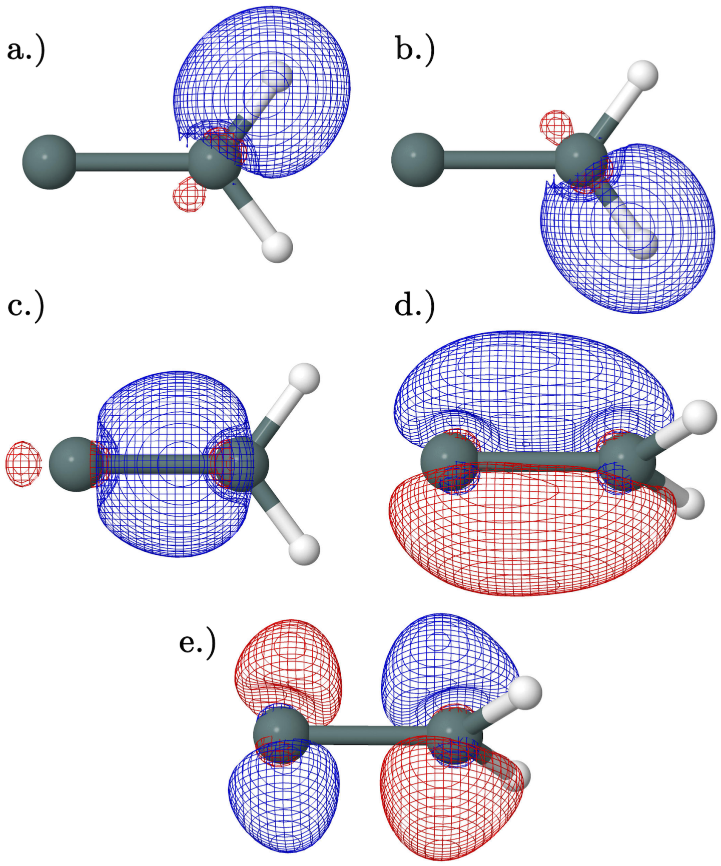

Figure S5: Natural bonding orbitals obtained from NBO analysis of CCSD(T)/QZ geometry of the neutral vinylidene-like minimum: a-b.) Degenerate Sn-H bonding orbitals. c.) Sn-Sn σ character bonding orbital. d.) HOMO Sn-Sn π character bonding orbital. e.) LUMO Sn-Sn π character anti-bonding orbital.

S5.3.2 Cation

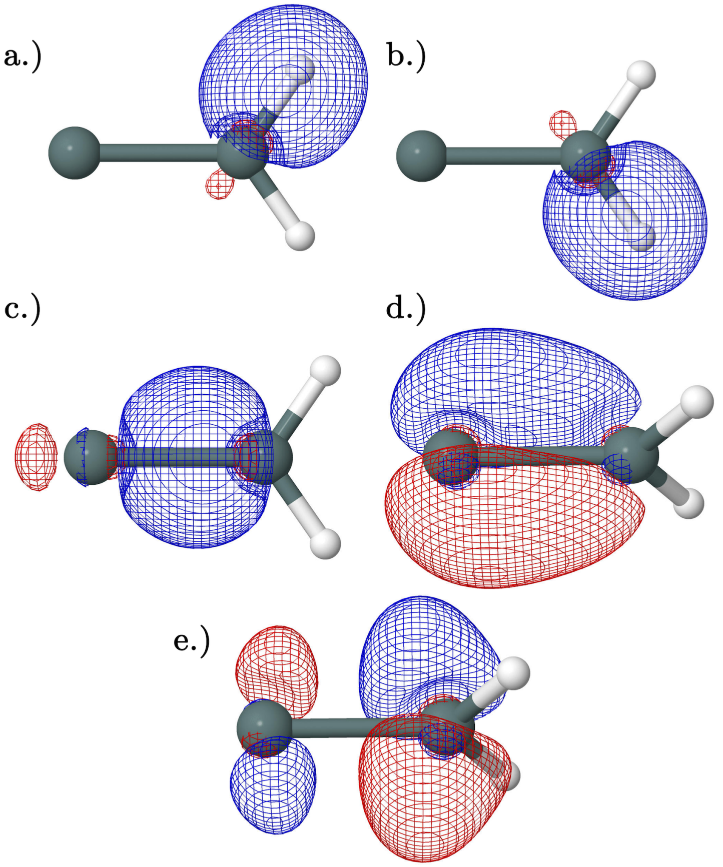

Figure S6: Natural bonding orbitals obtained from NBO analysis of CCSD(T)/QZ geometry of the cation vinylidene-like minimum: a-b.) Degenerate Sn-H bonding orbitals. c.) Sn-Sn σ character bonding orbital. d.) HOMO Sn-Sn π character bonding orbital. e.) LUMO Sn-Sn π character anti-bonding orbital.

S5.4 Nonplanar Trans

S5.4.1 Neutral

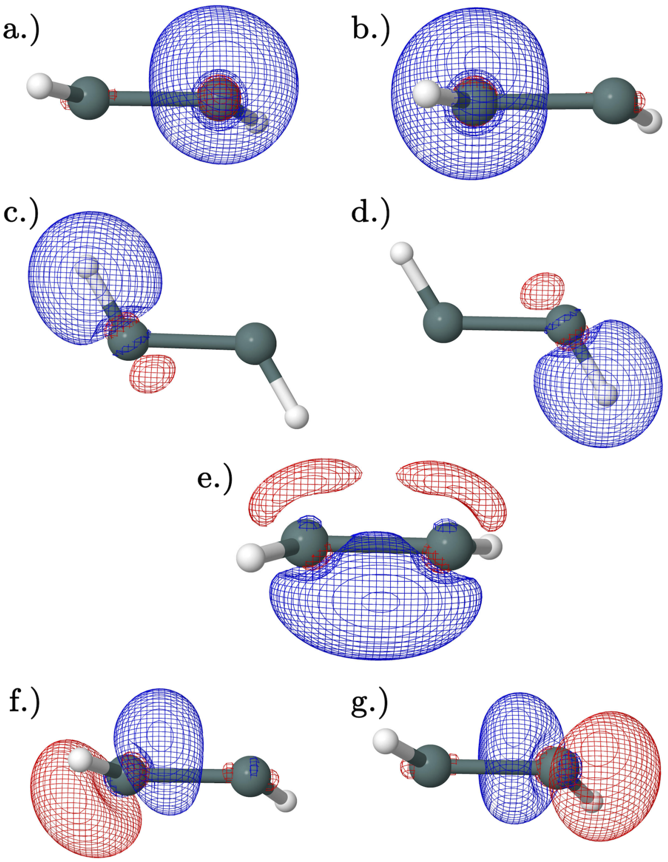

Figure S7: Natural bonding orbitals obtained from NBO analysis of CCSD(T)/QZ geometry of the neutral nonplanar trans transition state: a-b.) Degenerate Sn lone pair orbitals. c-d.) Degenerate Sn-H bonding orbitals. e.) HOMO Sn-Sn bonding orbital with primarily π character. f.) LUMO g-h.) Important bonding interaction orbitals. Orbitals a and g and orbitals b and h have a second order perturbation interaction energy of 92.87 kcal mol^{-1}.

S5.5 Planar Dibridged

S5.5.1 Neutral

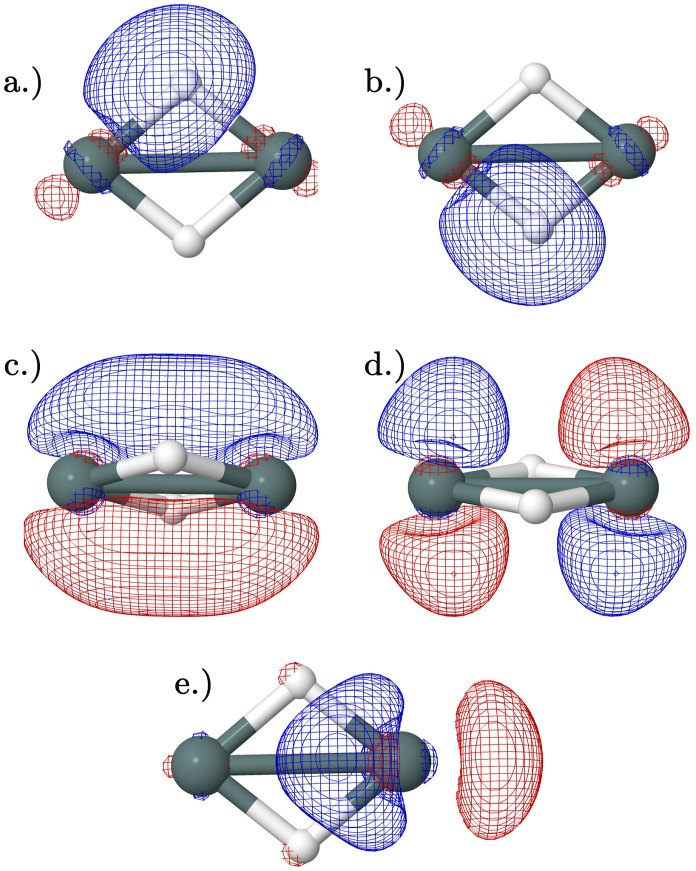

Figure S8: Natural bonding orbitals obtained from NBO analysis of CCSD(T)/QZ geometries of the neutral planar dibridged transition state: a-b.) Degenerate Sn-H bonding orbitals c.) HOMO Sn-Sn bonding orbital with primarily π character. d.) LUMO Sn-Sn π character antibonding orbital. e.) Unoccupied interacting orbital. Orbitals a and e as well as orbitals b and e have a second order perturbation interaction energy of 152.59 kcal mol^{-1}.

S5.5.2 Cation

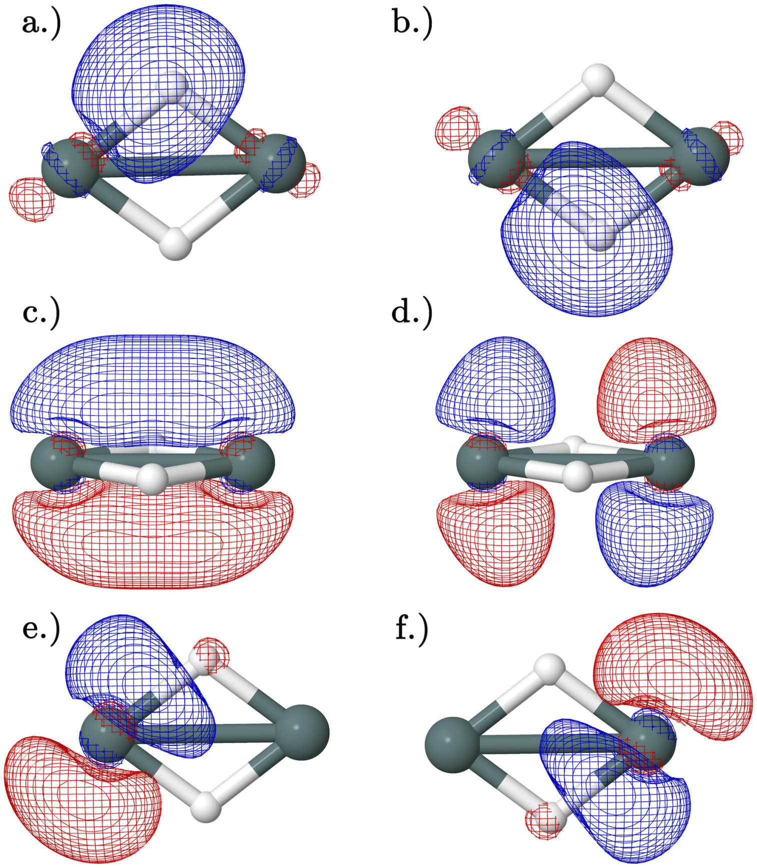

Figure S9: Natural bonding orbitals obtained from NBO analysis of CCSD(T)/QZ geometries of the cation planar dibridged transition state: a-b.) Degenerate Sn-H bonding orbitals c.) HOMO Sn-Sn bonding orbital with primarily π character. d.) LUMO Sn-Sn π character antibonding orbital. e-f.) Unoccupied interacting orbitals. Orbitals a and e as well as orbitals b and f have a second order perturbation interaction energy of 63.40 kcal mol^{-1}.

S5.6 Planar Trans

S5.6.1 Neutral

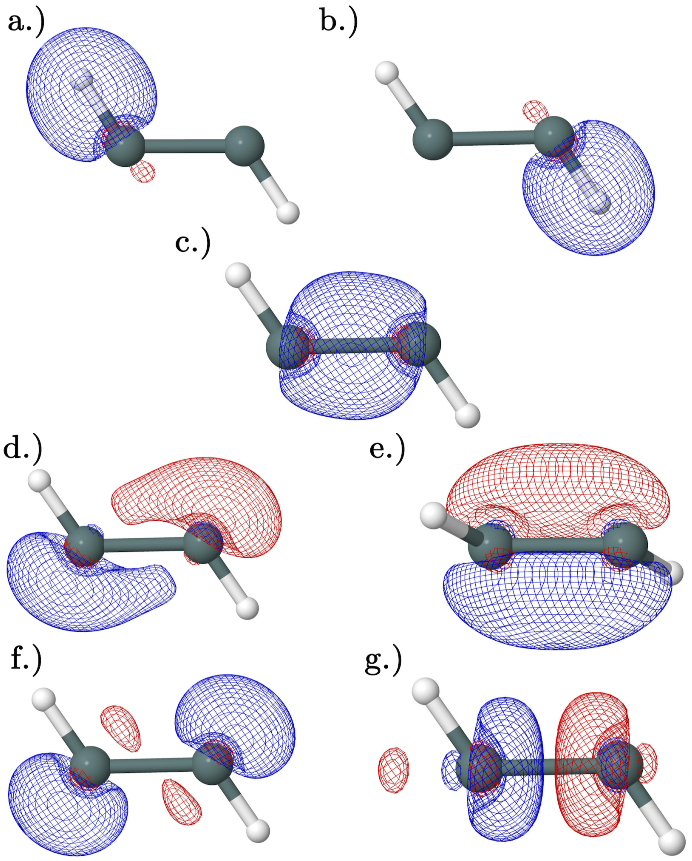

Figure S10: Natural bonding orbitals obtained from NBO analysis of CCSD(T)/QZ geometry of the neutral planar trans transition state: a-b.) Degenerate Sn-H bonding orbitals. c.) Sn-Sn bonding orbital with primarily σ character. d.) Sn-Sn bonding orbital with primarily π character. e.) HOMO Sn-Sn bonding orbital with primarily π character. f.) LUMO g.) Important bonding interaction orbital. Orbitals c and f have a second order perturbation interaction energy of 102.52 kcal mol^{-1}, and orbitals d and g have a second order perturbation interaction energy of 87.47 kcal mol^{-1}.

S5.5.2 Cation

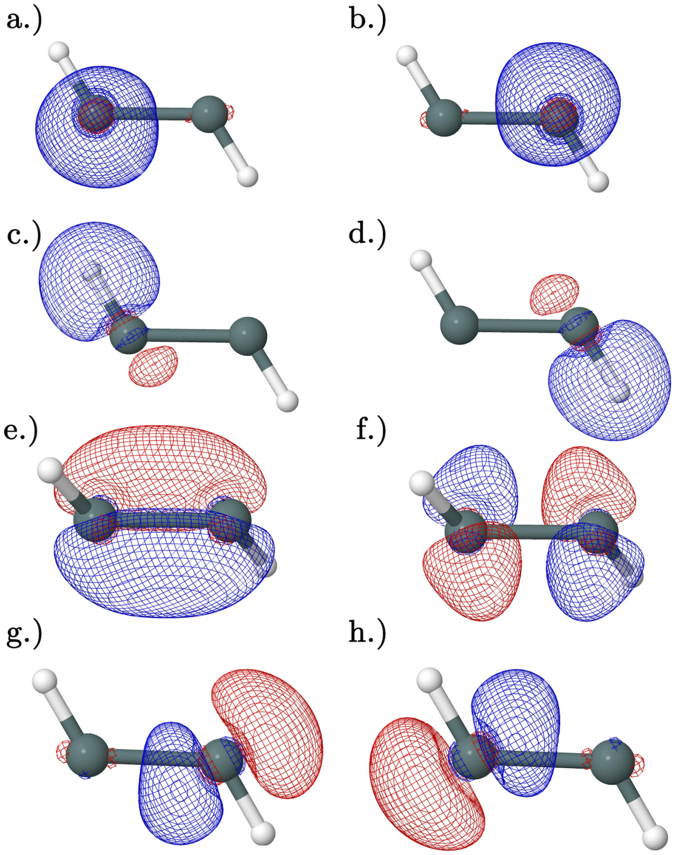

Figure S11: Natural bonding orbitals obtained from NBO analysis of CCSD(T)/QZ geometry of the cation planar trans transition state: a-b.) Degenerate Sn lone pair orbitals. c-d.) Degenerate Sn-H bonding orbitals. e.) HOMO Sn-Sn bonding orbital with primarily π character. f.) LUMO g-h.) Important bonding interaction orbitals. Orbitals a and g and orbitals b and h have a second order perturbation interaction energy of 45.60 kcal mol^{-1}.

S5.7 Perptrans

S5.7.1 Cation

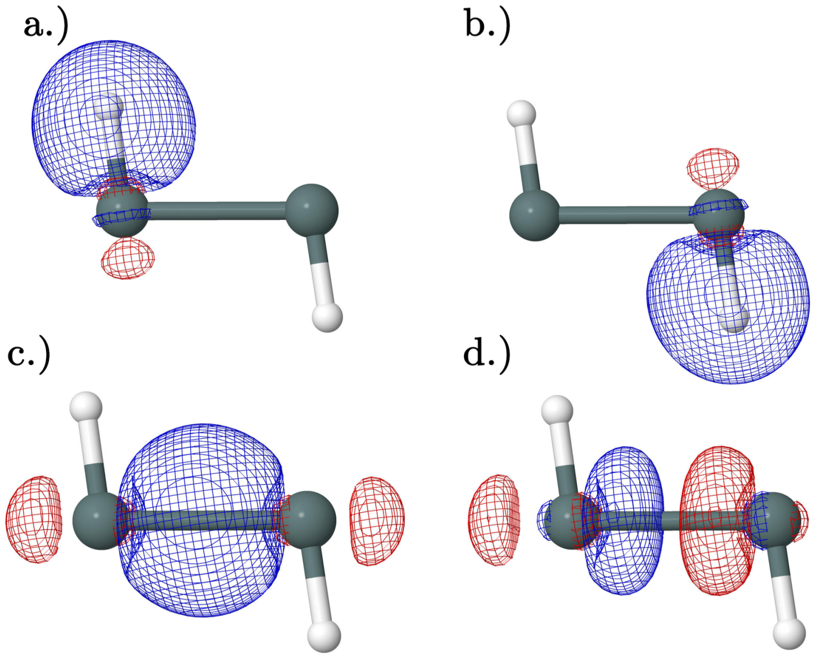

Figure S12: Natural bonding orbitals obtained from NBO analysis of CCSD(T)/QZ geometry of the cation perptrans transition state: a-b.) Degenerate Sn-H bonding orbitals. c.) SOMO Sn-Sn σ character bonding orbital. d.) LUMO Sn-Sn σ character anti-bonding orbital.

S5.8 Cis

S5.8.1 Neutral

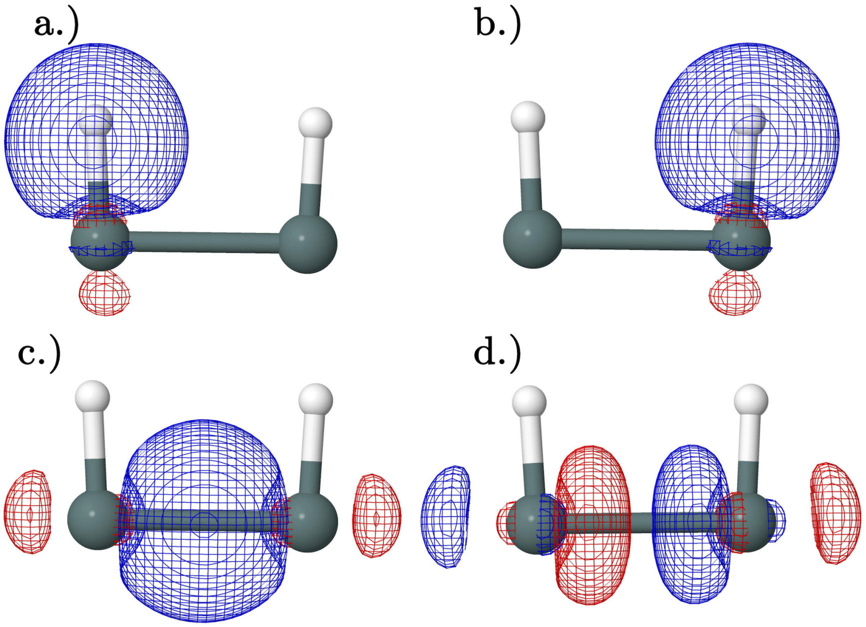

Figure S13: Natural bonding orbitals obtained from NBO analysis of CCSD(T)/QZ geometry of the neutral cis transition state: a-b.) Degenerate Sn-H bonding orbitals. c.) HOMO Sn-Sn σ character bonding orbital. d.) LUMO Sn-Sn σ character anti-bonding orbital.

S5.8.2 Cation

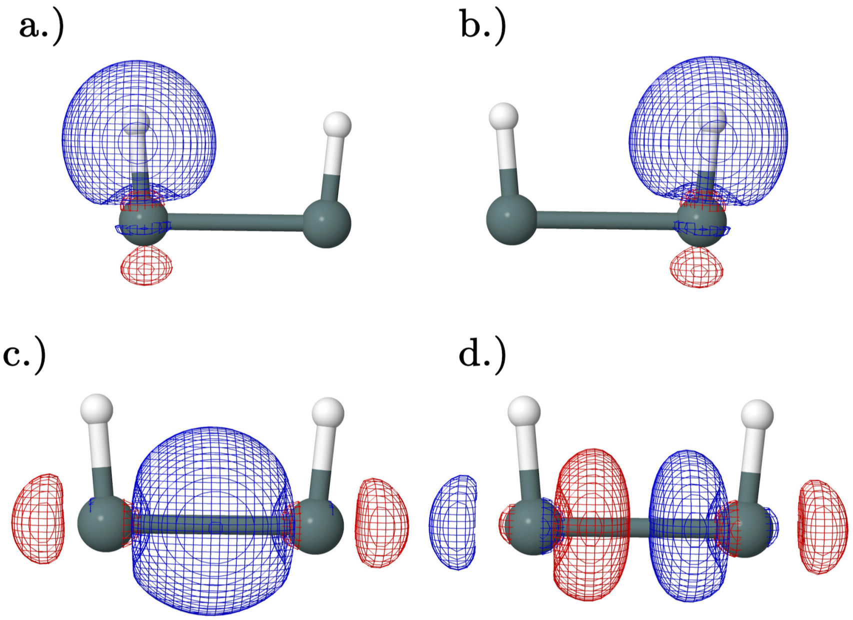

Figure S14: Natural bonding orbitals obtained from NBO analysis of CCSD(T)/QZ geometry of the cation cis transition state: a-b.) Degenerate Sn-H bonding orbitals. c.) HOMO Sn-Sn σ character bonding orbital. d.) LUMO Sn-Sn σ character anti-bonding orbital.

S5.9 Linear

S5.9.1 Neutral

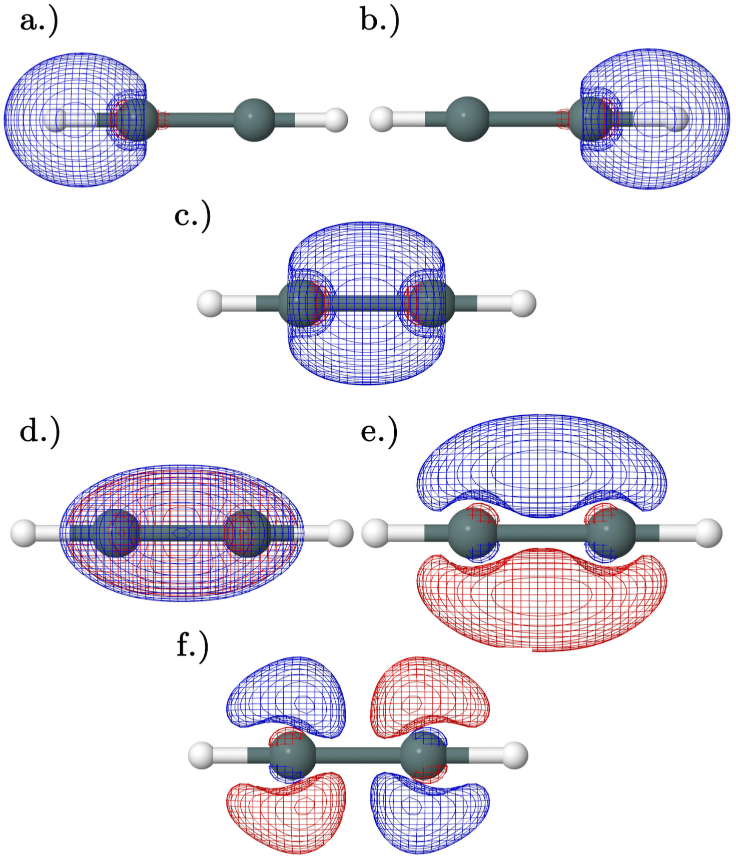

Figure S15: Natural bonding orbitals obtained from NBO analysis of CCSD(T)/QZ geometry of the neutral linear transition state: a-b.) Degenerate Sn-H bonding orbitals. c.) HOMO Sn-Sn σ character bonding orbital. d-e.) Degenerate HOMO Sn-Sn π character bonding orbitals. f.) LUMO Sn-Sn π character anti-bonding orbitals.

S5.9.2 Cation

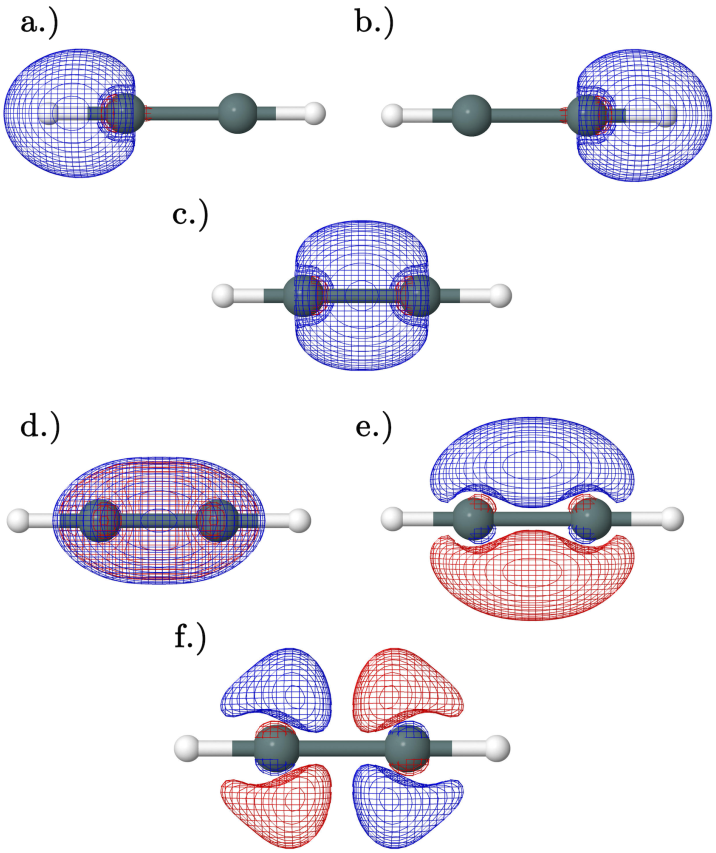

Figure S16: Natural bonding orbitals obtained from NBO analysis of CCSD(T)/QZ geometry of the cation linear transition state: a-b.) Degenerate Sn-H bonding orbitals. c.) HOMO Sn-Sn σ character bonding orbital. d-e.) Degenerate HOMO Sn-Sn π character bonding orbitals. f.) LUMO Sn-Sn π character anti-bonding orbitals.
